# Androgen-responsive FOXP4 is a target for endometrial carcinoma

**DOI:** 10.1038/s42003-024-06433-w

**Published:** 2024-06-18

**Authors:** Kayo Kayahashi, Mahadi Hasan, Anowara Khatun, Susumu Kohno, Jumpei Terakawa, Shin-ichi Horike, Natsumi Toyoda, Ayumi Matsuoka, Takashi Iizuka, Takeshi Obata, Masanori Ono, Yasunari Mizumoto, Chiaki Takahashi, Hiroshi Fujiwara, Takiko Daikoku

**Affiliations:** 1 Department of Obstetrics and Gynecology, Graduate School of Medical Sciences, Kanazawa University, Kanazawa, Japan; 2 Division of Animal Disease Model, Research Center for Experimental Modeling of Human Disease, Kanazawa University, Kanazawa, Japan; 3 Cancer Research Institute, Kanazawa University, Kanazawa, Japan; 4 Graduate School of Veterinary Science, Azabu University, Sagamihara, Japan; 5 Laboratory of Toxicology, School of Veterinary Medicine, Azabu University, Sagamihara, Japan; 6 Division of Integrated Omics Research, Research Center for Experimental Modeling of Human Disease, Kanazawa University, Kanazawa, Japan; 7 Department of Obstetrics and Gynecology, Toyama Prefectural Central Hospital, Toyama, Japan; 8 Department of Obstetrics and Gynecology, Tokyo Medical University, Nishi-Shinjuku, Japan; 9 Institute for Frontier Science Initiative, Kanazawa University, Kanazawa, Japan; 10 Ochi Yume Clinic, Nagoya, Aichi Japan

**Keywords:** Endometrial cancer, Oncogenesis

## Abstract

Although low estrogen is considered to suppress uterine endometrial carcinoma, the most cases occur in the postmenopausal stage. After menopause, the production of androgen level also declines. Therefore, to resolve the above enigma, we hypothesize that the postmenopausal decline of androgen is a trigger of its progression. In the present study, to validate this hypothesis, we examine the pathological roles of androgen/AR by analyzing clinical data, culturing endometrioid cancer cell lines, and using murine models. Clinical data show that androgen receptor (AR) expression and serum dihydrotestosterone (DHT) are associated with lower disease-free survival (DFS). DHT suppresses malignant behaviors in AR-transfected human endometrial cancer cells (ECC). In ovariectomized *Pten*^*ff*^*/PR*^*cre/+*^ mice, DHT decreases the proliferation of spontaneously developed murine ECC. In AR-transfected human ECC and *Pten*^*ff*^*/PR*^*cre/+*^ mice, DHT suppresses FOXP4 expression. FOXP4-overexpressed human ECC increases, while FOXP4-knocked-down ECC shows decreased malignant behaviors. DHT/AR-mediated ECC suppression is restored by FOXP4 overexpression. The high FOXP4 expression is significantly correlated with low postoperative DFS. These findings indicate that the androgen/AR system suppresses the malignant activity of endometrial carcinoma and that downstream FOXP4 is another target molecule. These findings will also impact developments in clinical approaches to elderly health.

## Abstract (web summary)

This study indicates that the androgen/AR system suppresses the malignant activity of endometrioid carcinoma through the downregulation of FOXP4 which will impact new developments in clinical approaches to elderly health.

## Introduction

Health care for postmenopausal women is one of the most important issues. To improve quality of life, hormone replacement therapy (HRT) is widely applied, but this therapy is considered to increase the risk of estrogen-dependent endometrial (endometrioid) carcinoma^1,2^.

Endometrioid carcinoma is considered to hormone-dependently develop and progress. Although a low estrogen status is believed to be advantageous in suppressing uterine cancer, most endometrioid carcinoma occurs in the postmenopausal elderly, who are in an inactive phase of life when serum estrogen levels are low^3–5^. However, there is currently no clear explanation for the mechanism by which somatic carcinoma develops during the inactive phase of the endometrium.

After menopause, the production of androgen also declines. Although details of the effects of male hormones on the uterus are still unknown, the possible involvement of androgen in the development of uterine cancer has been proposed since the androgen receptor (AR) is expressed in endometrioid carcinoma^6–8^. Some studies suggest an antagonistic role of androgen in the pathogenesis of endometrial carcinoma. Among male hormones, androstenedione and testosterone are aromatized by aromatase and can be converted to estrogen, but dihydrotestosterone (DHT) is not aromatized and exhibits the strongest effect as an androgen via AR. It was reported that proliferation of the human endometrial cancer cell line was inhibited by DHT^9^. It was also shown that candidates of AR target genes using the human endometrial cancer cell line, but the precise mechanisms of these molecules are not known^10^.

Based on the above background, we focused on the postmenopausal decline of androgen and hypothesized that this androgen reduction is a trigger of the postmenopausal progression of endometrioid carcinoma. In the present study, to validate this hypothesis, we examined the pathological roles of androgen/AR by analyzing clinical data, culturing endometrioid cancer cell lines, and using murine models: *Pten*^*ff*^*/PR*^*cre/+*^ mice lacking the *Pten* gene including the uterine stroma spontaneously develop endometrioid carcinoma^11^, and *Pten*^*ff*^*/LTF*^*cre/+*^ mice lacking the *Pten* gene only in epithelial cells develop hyperplasia^12^. We also explored downstream molecules of the androgen/AR pathway and examined the pathological role in the progression of endometrioid carcinoma.

## Results

### Analysis of the androgen/AR system in humans

**Fig. 1:**
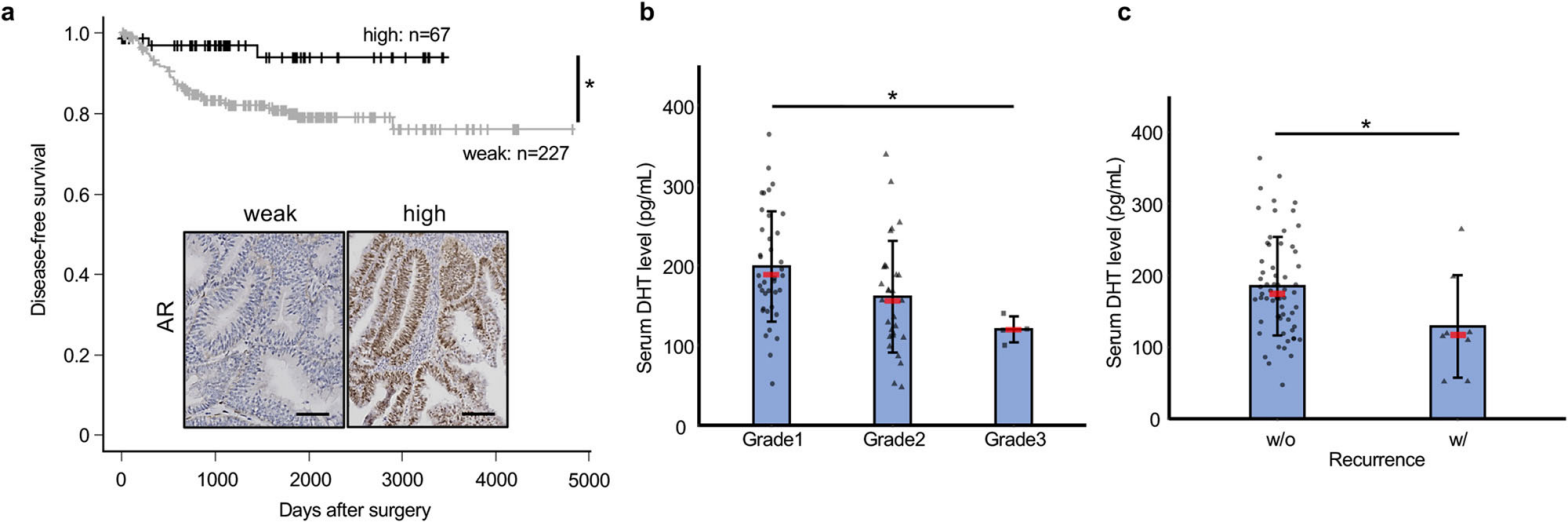
Analyses of the androgen/AR system in humans. **a** DFS rate. Bars show 100 µm. **b**, **c** Correlation between serum DHT levels and cancer grade (**b**) or recurrence (**c**). Statistical analysis was performed by the *χ*^2^ test (**a**), Kruskal–Wallis (**b**), and Mann–Whitney *U* (**c**) tests. Error bars represent standard deviation (**b**, **c**). Red cross bars show the median (**b**, **c**). *p < 0.05.

In human clinical specimens, low expression of AR was associated with lower DFS in patients with uterine endometrioid carcinoma (Fig. 1a). High expression of AR was significantly correlated with the grade, but not with myometrial invasion, lymph node metastasis, or distant site metastasis (Supplementary Table 1). The serum levels of DHT were lower in patients with Grade 3 or recurrence as compared with patients with Grade 1 or without recurrence, respectively (Fig. 1b, c). Although there are a few contradictory reports^13^, our findings indicate that AR expression is a promising clinical indicator of the prognosis.

### Analysis of the androgen/AR system in endometrial cancer cells and mouse models

**Fig. 2:**
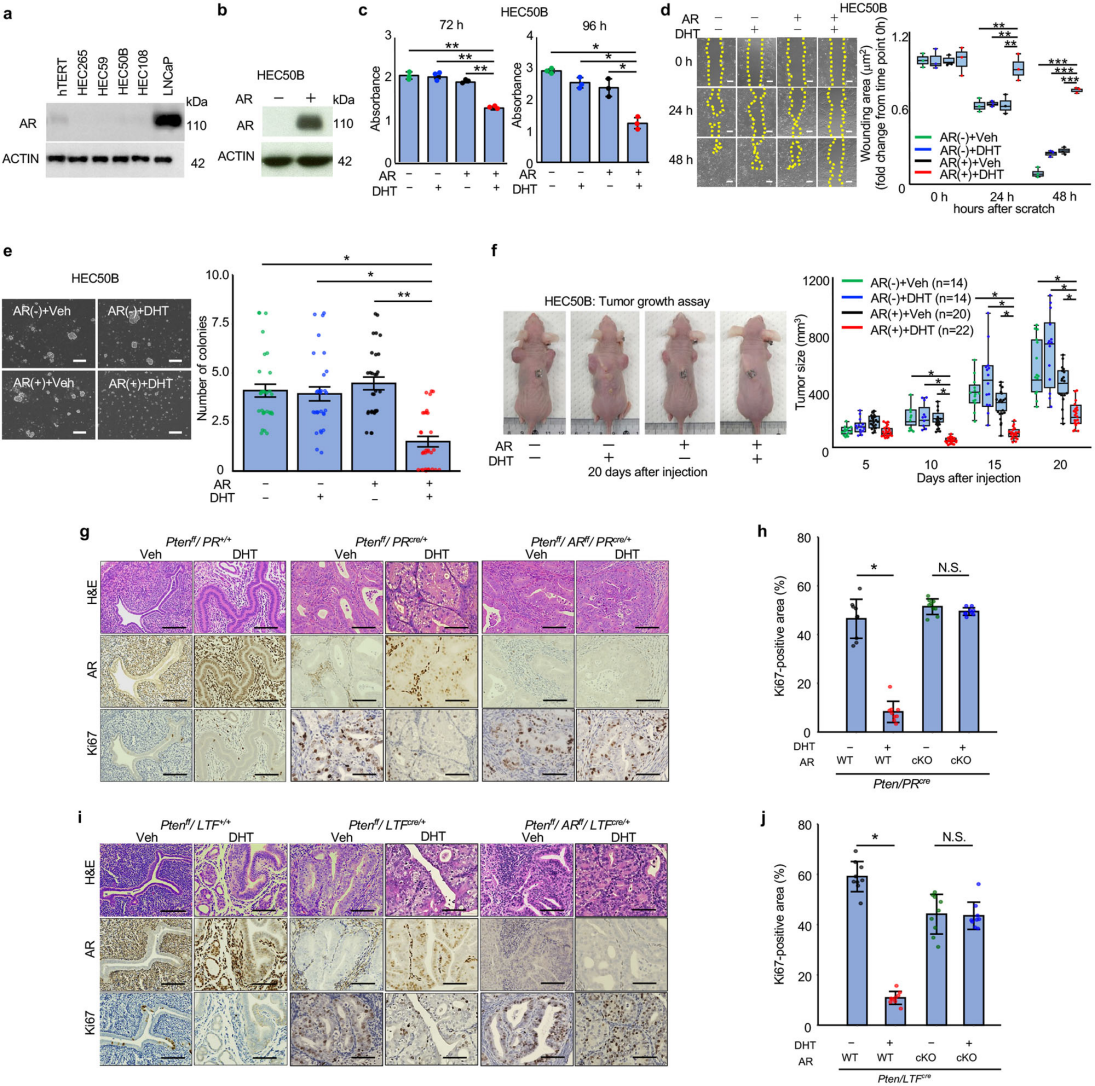
Analyses of the androgen/AR system in cell lines and mice models. **a** AR expression in cell lines. **b** HEC50B was transfected with AR overexpression vector (AR (+)). The 10 nM DHT treatment reduced cell viability (**c**), wound healing (*n* = 3) (**d**), and colony-forming ability (**e**) in AR(+)HEC50B cells. Bars show 200 µm (**d**, **e**). **f** DHT treatment reduced tumor growth of AR(+)HEC50B cells in the ovariectomized nude mice. **g**, **h** Histological uterine findings of the ovariectomized *Pten*^*ff*^*/PR*^*+/+*^, *Pten*^*ff*^*/PR*^*cre/+*^, and *Pten*^*ff*^*/AR*^*ff*^*/PR*^*cre/+*^ mice (*n* = 3/each group). **g** DHT promoted AR expression in *Pten*^*ff*^*/PR*^*+/+*^ and *Pten*^*ff*^*/PR*^*cre/+*^mice. Bar shows 200 µm. **h** DHT decreased the malignant cell proliferation in *Pten*^*ff*^*/PR*^*cre/+*^mice. In *Pten*^*ff*^*/AR*^*ff*^*/PR*^*cre/+*^ mice, DHT showed no effects on malignant cell proliferation. **i**, **j** Similar effects of DHT were observed in *Pten*^*ff*^*/LTF*^*cre/+*^ mice. Bar shows 200 µm. Statistical analysis was performed by the ANOVA followed by the Dunnett test (**c**–**f**), and Mann–Whitney *U* test (**h**, **j**). Error bars represent standard deviation (**h**, **j**) and standard error (**c**–**f**). *p < 0.05, **p < 0.01, ***p < 0.001.

AR expression was undetectable in the human ECC cell lines HEC50B, HEC59, HEC108, and HEC265, whereas it was detected in an immortalized human endometrial epithelial cell line, EM-E6/E7/hTERT cells^14^ and human prostate cancer cell, LNCaP (Fig. 2a). Then, we transfected HEC50B with AR (Fig. 2b). In AR-transfected HEC50B (AR_HEC50B) cells, DHT significantly decreased cell proliferation (Fig. 2c), delayed wound healing (Fig. 2d), and decreased the colony-forming ability (Fig. 2e). DHT also inhibited tumor formation of subcutaneously transplanted AR_HEC50B cells in ovariectomized nude mice (Fig. 2f).

We previously reported that *Pten*^*ff*^*/PR*^*cre/+*^ mice, which lack the *Pten* gene in both uterine epithelial and stromal cells, spontaneously develop endometrioid carcinoma^11^. In contrast, *Pten*^*ff*^*/LTF*^*cre/+*^ mice lacking the *Pten* gene only in epithelial cells develop hyperplasia, but not endometrioid carcinoma^12^. In this study, in ovariectomized wild-type (*Pten*^*ff*^*/PR*^*+/+*^) mice, DHT promoted AR expression in endometrial epithelial cells. Similarly, in ovariectomized *Pten*^*ff*^*/PR*^*cre/+*^ mice, AR expression in the spontaneously developed ECC was increased by DHT administration (Fig. 2g) and this treatment decreased the proliferation of ECC (Fig. 2g, h). In contrast, in ovariectomized *Pten*^*ff*^*/AR*^*ff*^*/PR*^*cre/+*^ mice lacking both *Pten* and *AR* genes, DHT showed no inhibitory effects on ECC proliferation (Fig. 2g, h). These findings indicate that DHT suppresses the progression of murine endometrioid carcinoma via the androgen/AR system. Similarly, in ovariectomized *Pten*^*ff*^*/LTF*^*cre/+*^ mice, DHT reduced the proliferation of atypical cells, but showed no inhibitory effects in *Pten*^*ff*^*/AR*^*ff*^*/LTF*^*cre/+*^ mice (Fig. 2i, j), suggesting that the androgen/AR system is also involved in the development of endometrial hyperplasia.

### Analysis of FOXP4 function as a downstream target of AR in the model system

**Fig. 3:**
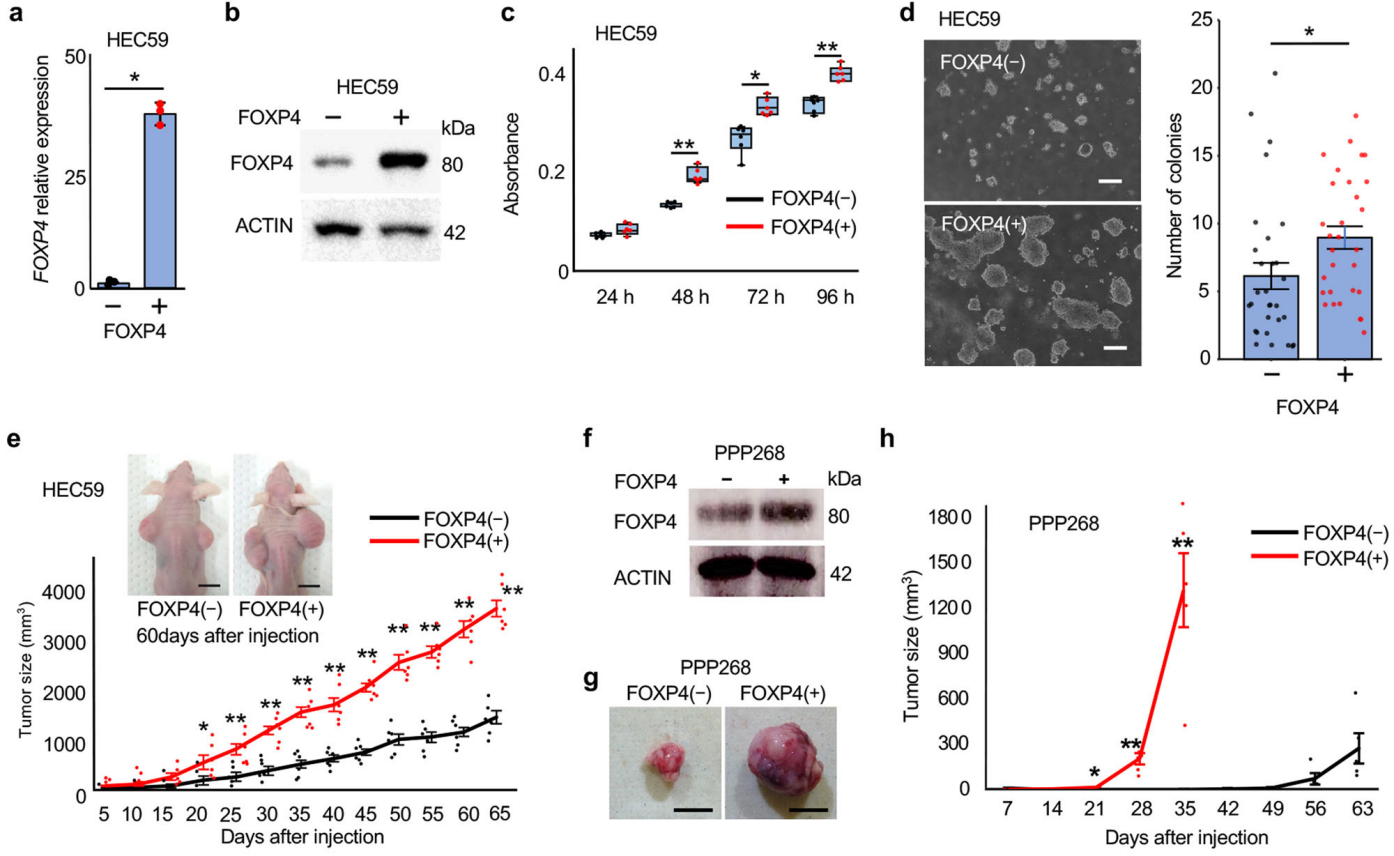
Analyses FOXP4 expression and its function by overexpression. **a**, **b** Expression levels of FOXP4 mRNA (**a**) and protein (**b**). HEC59 cells were transfected with a FOXP4 overexpression vector. Cell proliferation (*n* = 6) (**c**), colony-forming ability (**d**), and tumor growth (*n* = 6) (**e**) were promoted in FOXP4(+) HEC59 cells. Bars show 200 µm (**d**) and 1 cm (**e**). In FOXP4-overtransfected PPP268 cells (**f**), tumor growth was also promoted (*n* = 6) (**g**, **h**). Bars show 1 cm (**g**). Statistical analysis was performed by the Mann–Whitney *U* test (**a**, **c**–**e**, **h**). Error bars represent standard error. *p < 0.05, **p < 0.01.

Recently, we reported that forkhead box transcription factor P4 (FOXP4) is associated with the androgen/AR pathway in cervical intraepithelial neoplasia cells^15^. FOXP4 was expressed in human cell lines of ECC, HEC50B, HEC108, and HEC265 (Supplementary Fig. 1a), and its expression in AR_HEC50B cells was downregulated by DHT treatment in mRNA (Supplementary Fig. 1b) and protein (Supplementary Fig. 1c) levels. FOXP4 was expressed in the spontaneously developed endometrioid carcinoma of *Pten*^*ff*^*/PR*^*cre/+*^ mice (panel 3 in Supplementary Fig. 1d), and its expression was decreased by DHT treatment (panel 4 in Supplementary Fig. 1d). These suppressing effects were absent when AR was deficient (panels 5 and 6 in Supplementary Fig. 1d). A similar suppressing effect on FOXP4 expression by DHT was also observed in atypical endometrial epithelial cells of *Pten*^*ff*^*/LTF*^*cre/+*^ mice (panels 7–12 in Supplementary Fig. 1d). To determine whether FOXP4 is a downstream target molecule of the androgen/AR pathway in endometrial cancer, we performed a Chip-sequence assay and found that androgen/AR or its transcription complex bound to intron 1 of the h*FOXP4* coding region of AR_HEC50B cells (Supplementary Fig. 1e). Next, 3.2 kb of the AR-binding site (Supplementary Fig. 1f) of AR_HEC50B cells was deleted (Supplementary Fig. 1g) and the effect of DHT on FOXP4 expression was examined. By this treatment, the inhibitory effect of DHT on FOXP4 expression disappeared (Supplementary Fig. 1h), confirming that androgen/AR or its transcription complex regulates FOXP4 expressions by directly binding to the FOXP4 coding region. However, further investigation is required to determine whether FOXP4 transcription is controlled by AR itself binding to the genomic DNA or by a complex containing AR. FOXP4 was overexpressed in HEC59 cells, which showed weak FOXP4 expression in the wild-type (Fig. 3a, b). In FOXP4(+) HEC59 cells, cell proliferation (Fig. 3c) and colony-forming ability (Fig. 3d) were significantly increased. Tumor formation was also promoted when FOXP4(+) HEC59 cells were subcutaneously transplanted into nude mice (Fig. 3e). Similarly, malignant behaviors were promoted in FOXP4-overexpressed HEC108 cells (Supplementary Fig. 2). In addition, when a murine endometrioid carcinoma-derived cell line, PPP268, was transfected with FOXP4 (Fig. 3f) and subcutaneously allografted in C57BL/6 N mice, tumor formation was significantly promoted as compared with wild-type PPP268 (Fig. 3g, h). This result indicates that FOXP4 promotes tumor formation even when recipient mice have normal immune functions.

**Fig. 4:**
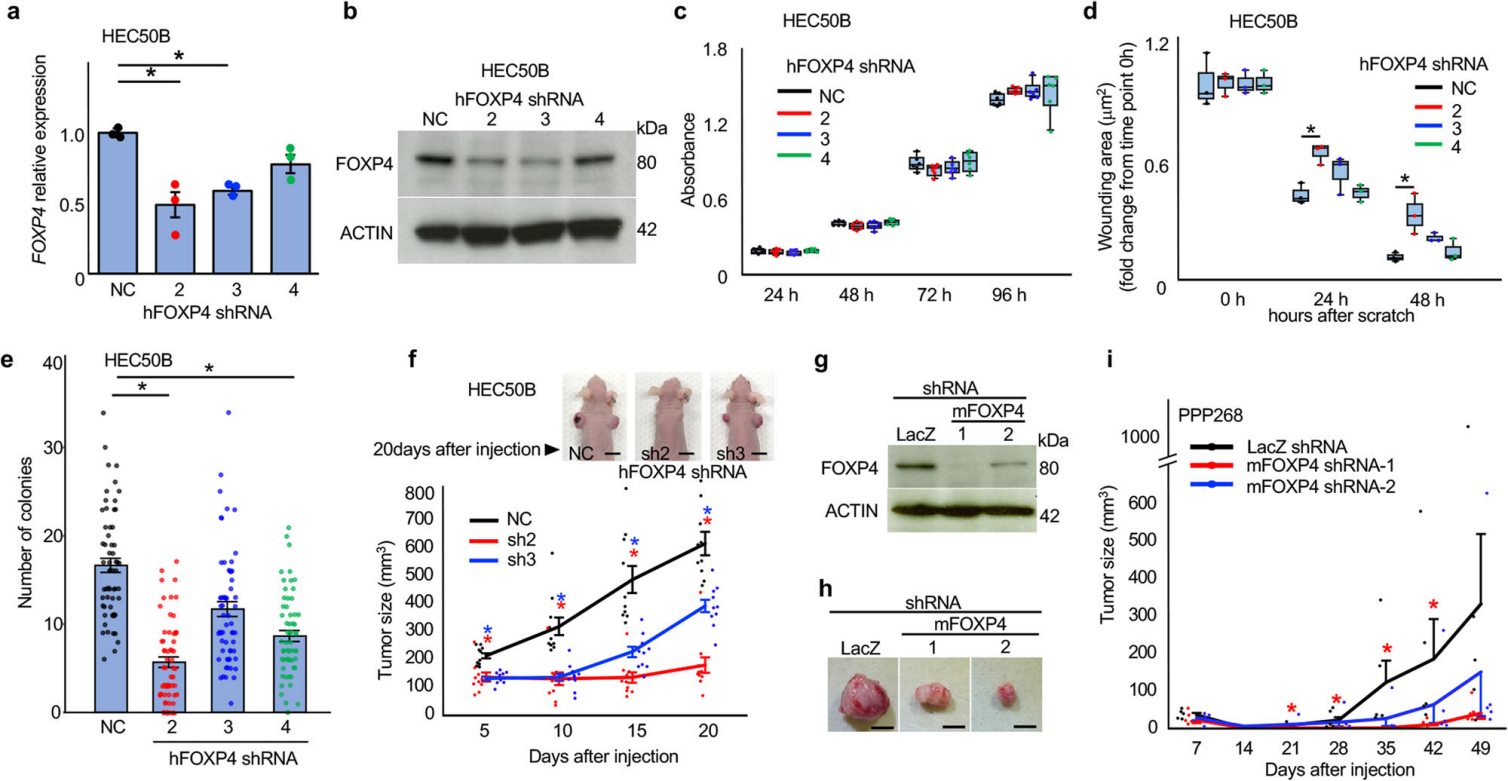
Analyses FOXP4 function by knockdown. FOXP4 in HEC50B cells was downregulated by shRNA (**a**, **b**). Although cell proliferation was not changed (*n* = 6) (**c**), wound-healing ability (*n* = 3) (**d**), colony-forming ability (**e**), and tumor growth (*n* = 10) (**f**) were decreased by FOXP4 knockdown. Bars show 1 cm (**f**). FOXP4 in PPP268 cells was also downregulated by shRNA (**g**). Tumor growth of FOXP4(–) PPP268 cells was decreased (*n* = 6) (**h**, **i**). Bars show 1 cm (**h**). Statistical analysis was performed by the ANOVA followed by the Dunnett test (**a**, **c**–**f**). Error bars represent standard error. *p < 0.05.

Then, FOXP4 expression in HEC50B cells was knocked down by shRNA (Fig. 4a, b). Although downregulation of FOXP4 expression did not change proliferation (Fig. 4c), this treatment significantly delayed wound healing (Fig. 4d) and decreased the colony-forming ability (Fig. 4e). Tumor formation was also inhibited when FOXP4-downregulated HEC50B cells were subcutaneously transplanted into nude mice (Fig. 4f). We further knocked down FOXP4 in HEC59, HEC265, and HEC6 cell lines. Similar changes in malignant behaviors were observed (Supplementary Figs. 3–5). When FOXP4-downregulated PPP268 cells (Fig. 4g) were subcutaneously allografted in C57BL/6 N wild-type mice, tumor formation was significantly reduced (Fig. 4h, i).

We further examined whether FOXP4 overexpression restores the DHT/AR-mediated suppression of ECC. We used FOXP4-mycflag or empty vector to transfect AR_HEC50B cells and created stable cell lines. DHT treatment decreased endogenous FOXP4 but not exogenous FOXP4 (FOXP4-mycflag) expression (Supplementary Fig. 6a). Regarding cell proliferation, DHT/AR-mediated suppression was partially restored by exogenous FOXP4 (Supplementary Fig. 6b). The delayed wound healing (Supplementary Fig. 6c) and decreased colony-forming ability (Supplementary Fig. 6d) induced by DHT/AR were completely reversed by exogenous FOXP4. These results suggest that FOXP4 mainly regulates cell migration, cell-cell interactions, and anchorage-independent growth.

### Analysis of FOXP4 function as a downstream target of AR in humans

**Fig. 5:**
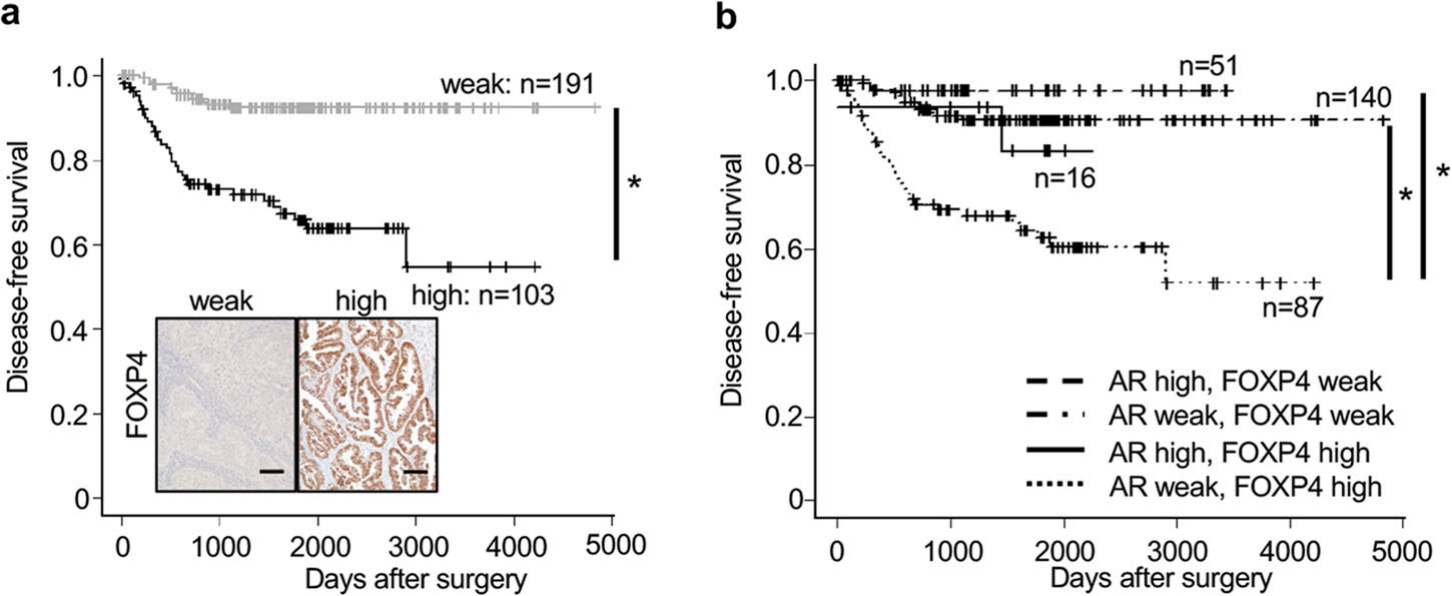
Analyses FOXP4 and AR in human. **a** Correlation between FOXP4 expression and DFS rate. Bar shows 100 µm. **b** Correlation between FOXP4/AR expression and DFS rate. Statistical analysis was performed by *χ*^2^ test (**a**, **b**). Error bars represent standard error. *p < 0.051.

The above results suggest that the progression of endometrioid carcinoma can be controlled by stimulating an androgen/AR system and/or suppressing its downstream molecular target, FOXP4. To support this, DFS associated with endometrioid carcinoma is significantly reduced in patients with high FOXP4 expression (Fig. 5a). The combination of low expression of AR and high expression of FOXP4 was particularly associated with low DFS (Fig. 5b). FOXP4 expression is inversely correlated with AR expression (Supplementary Table 1). Multivariate analysis revealed that high FOXP4 expression was an independent prognostic factor (Supplementary Table 2).

## Discussion

As a major change in elderly women, this study focused on the significance of physiological changes in male hormones, which have been studied secondarily to female hormones. The results of the present study support our initial hypothesis that the postmenopausal decline of androgen is a risk factor for endometrial cancer.

Clinical data showed that low expression of AR was associated with lower postoperative disease-free survival in patients with uterine endometrioid carcinoma. Although there are a few contradictory reports^13^, our findings are compatible with most previous studies, indicating that AR expression is a clinical indicator of the prognosis associated with uterine endometrioid carcinoma^16–18^. Although these results suggest that androgen has suppressing effects on endometrial cancers, experimental validation has not been obtained^8^.

Therefore, we examined the in vitro effects of androgen on endometrial cancer behaviors using HEC50B cells, which are derived from human endometrioid carcinoma. In AR-transfected HEC50B cells, DHT induced a significant decrease in cell proliferation, wound healing, colony formation, and tumor formation, indicating that androgen suppresses the progression of endometrioid cancer cells in vitro. To confirm the effects of androgen on endometrioid carcinoma progression in vivo, we conducted animal experiments using uterus-specific PTEN-deficient mice that spontaneously developed endometrial carcinoma of the uterus, and found that DHT decreased the proliferation of the spontaneously developed endometrioid carcinoma in ovariectomized *Pten*^*ff*^*/PR*^*cre/+*^ mice. In contrast, DHT showed no effects on cell proliferation in murine endometrioid carcinoma in the ovariectomized AR-lacking *Pten*^*ff*^*/AR*^*ff*^*/PR*^*cre/+*^ mice, indicating that the suppressing effect of DHT is achieved via the androgen/AR system.

To further elucidate the pathological roles of androgen/AR in endometrial carcinogenesis, this study employed unique *Pten*^*ff*^*/LTF*^*cre/+*^ mice that spontaneously develop endometrial hyperplasia. Using this model, we analyzed the contribution of androgenic hormones to the development of endometrial hyperplasia. In the ovariectomized *Pten*^*ff*^*/LTF*^*cre/+*^ mice, DHT inhibited the proliferation of atypical endometrial cells. In contrast, with the lack of AR in epithelial cells, DHT showed no effects on cell proliferation in the ovariectomized *Pten*^*ff*^*/AR*^*ff*^*/LTF*^*cre/+*^ mice. This suggests that the androgen/AR system is also involved in the development of endometrial hyperplasia.

Until now, the molecular biological mechanism of the androgen/AR system for the development and progression of uterine cancer has remained unclear^7,8^. To the best of our knowledge, this study revealed that FOXP4 is a downstream factor of the androgen/AR system in human endometrioid cancer cells for the first time. Using both FOXP4-overexpressed HEC59 and 108 cells and FOXP4-downregulated HEC50B, 265, and 6 cells, FOXP4 was shown to activate malignant behaviors of endometrioid cancer cells in vitro and in vivo. Using a FOXP4-overexpressed murine endometrial carcinoma-derived cell line, tumor formation was significantly promoted when they were subcutaneously transplanted into recipient mice of the same inbred strain with normal immune functions. Additionally, androgen/AR-mediated suppression of ECC was restored by exogenous FOXP4 expression. These results indicate that the progression of endometrioid carcinoma can be controlled by suppressing the expression of FOXP4. Furthermore, clinical data concerning the expression profiles of FOXP4 and AR in endometrioid carcinoma support our experimental conclusion that the progression of endometrioid carcinoma can be controlled by stimulating an androgen/AR system and/or suppressing its downstream molecular target, FOXP4.

To the best of our knowledge, our findings will have a strong impact on bringing about new developments in clinical approaches to elderly health, especially in other sex hormone-dependent diseases such as breast and prostate cancers. The basic concept that climacteric hormonal changes may become a risk factor for hormone-dependent diseases should be re-evaluated in the future. This study showed that the supplemental administration of androgen inhibited cell proliferation in the region of endometrial hyperplasia in ovariectomized uterus-specific *Pten*-deleted mice, suggesting the promising role of androgen in the protection of endometrial carcinogenesis against hyperplasia. This means that androgen, especially DHT, treatment is a potential preventive and/or therapeutic strategy for endometrial carcinoma. In addition, to improve the quality of life of elderly women, any new HRT strategy for postmenopausal women should also be evaluated from the perspective of the additional administration of androgen or favorable use of progestin that additionally has androgenic effects.

This study had several limitations. First, our in vivo data are restricted to animal experiments. Although several studies reported that there were no adverse changes in the endometrium from patients who used gender-affirming testosterone therapy and underwent hysterectomy^19^, clinical data should be further analyzed to clarify the preventive roles of androgens against endometrial carcinogenesis. Second, although murine experiments suggest that DHT can enhance AR expression in ECC, endometrioid carcinoma associated with a poor prognosis showed a low level of AR expression. There is a report that the short poly-glutamine polymorphism (AR-Q_S_, <20) in human AR exon 1 is associated with lower overall survival of patients with endometrial cancers compared with long AR-Q (AR-Q_L_, 20<)^20^. In the endometrial cancer cell, AR-Q_S_ but not AR-Q_L_ interacts with the anyl hydrocarbon receptor (AhR) bound to benzo [a] pyrene, an environmental factor, and enhances cancer growth. In the present study, we utilized the human AR-Q_L_ (23 Q) construct to overexpress AR and suppress cancer growth through decreasing FOXP4 expression. Further investigation is required to examine whether AR-Q_S_/AhR affects FOXP4 expression. In human ECC cells, AR could not be detected (Fig. 2a). Furthermore, DHT could not enhance AR expression in HEC50B, which has an AR mutation(V714A) (Supplementary Fig. 1c and Supplementary Table 3). Thus, the poly-glutamine polymorphism and mutation status of AR should be examined in clinical samples to assess whether DHT can be used for treatment. In addition, clinically available agents, which can alternatively regulate the downstream pathway of the androgen/AR system, should be explored in the future. From the present study, FOXP4 is one of the candidates for clinically available agents as a downstream target of androgen/AR. However, there should be other factors to regulate FOXP4 expression since FOXP4 levels vary among human ECCs regardless of the presence of AR mutations (Supplementary Fig. 1a and Supplementary Table 3). For details, we need further studies.

In conclusion, this study showed that DHT suppresses the malignant activity of endometrioid carcinoma via the androgen/AR system. To the best of our knowledge, this study also suggests that FOXP4 is a new molecular target that regulates the progression of endometrioid carcinoma. These findings will contribute to developing a promising strategy to improve the quality of life of elderly female patients.

## Materials and methods

### Patients and tissue samples

We recruited 294 patients with endometrial carcinoma who underwent surgery from 2008 to 2019 at Kanazawa University Hospital, Japan. We utilized 294 formalin-fixed paraffin-embedded blocks of endometrial cancer and 72 plasma samples collected from patients with endometrial carcinoma. The study protocol was approved by the Medical Ethics Committee of Kanazawa University, and preoperative informed consent was obtained from the patients (approved no. 3346-1). All ethical regulations relevant to human research participants were followed.

### Mice

Six-week-old Balb/c-nu/nu female mice were purchased from SLC (Japan). Six-week-old C57BL/6 N females were purchased from The Jackson Laboratory (Japan). C57BL/6-background *Pten*^ff^/ *p53*^ff^/ *PR*^cre/+^ mice were kindly provided by Dr. Adrian Erlebacher (University of California San Francisco), and *Pten*^ff^/ *PR*^cre/+^ mice were established from *Pten*^ff^/ *p53*^ff^/ *PR*^cre/+^ mice crossed with C57BL/6 N females^21^. C57BL/6-background *Pten*^ff^/ *LTF*^cre/+^ mice were generated by crossing with C57BL/6 N mice for at least six generations^12^ *Pten*^ff^/ AR^ff^/ *PR*^cre/+^ and *Pten*^ff^/ AR^ff^/ *LTF*^cre/+^ mice were generated by crossing *AR*^ff^ mice^22^ with *Pten*^ff^/ *PR*^cre/+^ or *Pten*^ff^/ *LTF*^cre/+^ mice, respectively. All mice used in this study were housed in the Institute for Experimental Animals at Kanazawa University according to institutional guidelines for the care and use of laboratory animals. All protocols were reviewed and approved by the Institutional Animal Care and Use Committee, Kanazawa University (approval No. AP-204134). We have complied with all relevant ethical regulations for animal use.

### Cell lines

The human endometrial carcinoma cell lines, HEC265, HEC59, HEC50B, and HEC108, generated by Dr. Hiroyuki Kuramoto were kindly provided by Dr. Katsutoshi Oda (Graduate School of Medicine, Tokyo University)^23^. These cell lines were cultured with EMEM containing 10% FBS, penicillin, and streptomycin. The normal human endometrial glandular epithelial cell line (EM-E6/E7/hTERT) was cultured with DMEM/HamF12 containing 10% FBS penicillin, and streptomycin^14^. AR and FOXP4 mutations in these cell lines were identified from cbioportal (Supplementary Table 3). The human AR-positive prostate cancer cell line, LNCaP (RRID: CVCL_0395), was gifted by Dr. Atsushi Mizokami (Department of Urology, Graduate School of Medical Sciences, Kanazawa University). This cell line was cultured with DMEM containing 5% FBS penicillin and streptomycin. All human cell lines were authenticated by STR profiling. The mouse endometrial cancer cell line, PPP268, was established from the uterus of a 1-month-old *Pten*^f/f^ /*p53*^f/f^/ *PR*^cre/+^ female mouse. In brief, the uteri were slit longitudinally and cut into 2 to 3 mm in length. After washing several times with phenol red-free Hank’s Balanced Salt Solution (Sigma-Aldrich, St. Louis, MO) with 100 μg/mL of streptomycin, 100 U/mL of penicillin, and 2.5 μg/mL of amphotericin B (FUJIFILM Wako Pure Chemical Corporation, Japan. Cat no. 161-23181), these pieces were digested with 6 mg/mL of dispase (Life Technologies) and 25 mg/mL pancreatin (Sigma-Aldrich) for 1 hour at 4 °C, 1 hour at room temperature, and 10 min at 37 °C. After this process, digestion was stopped by adding Hank’s Balanced Salt Solution containing 10% FBS. The sheet of luminal epithelial cells dislodged by thoroughly mixing with a 25 ml pipet were collected by centrifuge and plated on the dish coated with Matrigel, which was diluted 4 times with PBS (Corning Inc, NY, cat no. 356237). PPP268 cells were cultured with DMEM/F12 including 10% FBS and antibiotics. Mycoplasma infections were regularly monitored, and all experiments were performed under mycoplasma-free conditions.

### Immunohistochemical staining and scoring

Immunohistochemical staining with 4 μm sections of human specimens was performed using the avidin–biotin–peroxidase complex method according to the manufacturer’s instructions (VECTASTAIN ABC Kit, Vector Laboratories, USA). After deparaffinization and antigen-retrieval, the slides were incubated overnight at 4 °C with one of the primary antibodies: anti-AR rabbit monoclonal antibody (1:200, clone SP107, RRID: AB_2537931, ab105225, Abcam, USA), anti-FOXP4 rabbit polyclonal antibody (1:200, HPA007176, Sigma, USA), or anti-Ki67 rabbit monoclonal antibody (1:200, clone sp6, RM-9106, Thermo Fisher, USA). Hematoxylin was used for counterstaining. Expression levels of AR and FOXP4 in malignant cells were assessed by the J score, which represents the percentage of immunoreactive cells. Each sample was divided into two groups for AR and FOPX4: High and Low. An AR score of 80 or higher and FOXP4 score of 50 or higher were classified as High. An AR score of 79 or lower and FOXP4 score of 49 or lower were classified as Low.

Immunohistochemical staining with 5 μm sections of mouse specimens was performed using the streptavidin–biotin–peroxidase (Jackson ImmunoResearch, USA). After deparaffinization and antigen-retrieval, the slides were incubated overnight at 4 °C with one of the primary antibodies: anti-AR rabbit monoclonal antibody (1:50, clone SP107, RRID: AB_2537931, ab105225, Abcam, USA), anti-FOXP4 rabbit polyclonal antibody (1:500, HPA007176, Sigma, USA), or anti-Ki67 rabbit monoclonal antibody (1:200, clone sp6, RM-9106, Thermo Fisher, USA). The signal was visualized with DAB (Fujifilm Wako Pure Chemical Corporation, Japan). Hematoxylin was used for counterstaining. Total nuclei and Ki67-positive nuclei in the epithelium were counted in three independent areas of three sections.

### ELISA

Serum DHT concentrations were measured by the ELISA kit (DHT, ELISA kit, ABV Abnova, cat no. KA1886, Taiwan) following the manufacturer’s instructions. Standard, control, and samples were added 50 µL to each well of the microplate. After adding DHT-HRP to each well, the microplate was shaken slowly for 10 sec and incubated at room temperature for 1 h. Each well was washed three times with 300 µL of wash buffer, and tetramethylbenzidine was added 150 µL to each well. The microplate was shaken slowly for 10 sec, then incubated at room temperature for 15 min. The absorbance was measured at 450 nm on a microtiter plate reader (iMark^TM^ microplate reader, BioRad, USA). A set of standards is used to plot a standard curve from which the amount of DHT in samples and controls can be directly read. Each sample was set up as a duplicate.

### Western blot analysis

Protein lysates were extracted with RIPA buffer. After measuring the concentration with the DC^TM^ protein assay kit (BioRad cat no. 5000111, USA), 25–30 μg of each protein lysate were electrophoresed on SDS-PAGE gels and then transferred to PVDF membranes. The transferred membranes were incubated with primary antibody against AR (1:1000, clone SP107, RRID: AB_2537931, ab105225, Abcam, USA, or rabbit polyclonal antibody, which was kindly gifted from Dr. Mizokami), FOXP4 (1:1000, RRID: AB_2262825, 16772-1-AP, Proteintech Group Inc., USA), and β-Actin (1:5000, RRID: AB_630835, C-11, Santa Cruz Biotechnology, USA) overnight at 4 °C. After incubation with secondary horseradish peroxidase (HRP)-conjugated antibody (Jackson ImmunoResearch), the blots were visualized using an enhanced chemiluminescence system and ECL Western Blotting Detection Reagents (GE Healthcare, USA).

### Quantitative real-time PCR analysis

Total RNA was extracted from clinical samples and the cell line using the RNeasy Mini kit (Qiagen, Germany) according to the manufacturer’s instructions. The RNA was reverse transcribed to cDNA using Superscript II (Invitrogen, USA). GAPDH was used as an internal control. The cDNA was amplified using specific primers (Supplementary Table 4). The conditions for PCR cycling were 94 °C for 120 sec and 40 cycles of 10 sec at 98 °C, followed by an annealing and extension step at 60 °C for 30 sec. Quantitative real-time PCR (qPCR) analysis was performed using the Mx3000P real-time PCR system (Agilent Stratagene, USA), according to the manufacturer’s instructions.

### AR and FOXP4 overexpression in cell lines

The empty vector (pCAGIPuro) and a human AR overexpression vector were described in a previous paper^15^. The protein-coding sequences of human and mouse FOXP4 to subclone into the pCAGIPuro vector were amplified by PCR using primers (Supplementary Table 4). Cells were seeded in 6-well plates at 5 × 10^5^ cells per well. After overnight culture, vectors were employed to transfect cells using GenomeOne GX (Ishihara Sangyo Kaisha, Ltd., Japan) and KALA amphipathic peptide (Cosmo Bio Co., LTD., Japan) according to the manufacturer’s protocol. At 48 h after transfection, 5 μg/mL puromycin (Thermo Fisher Scientific, USA) was added to the cells for 7 days to establish stable cell lines. The protein-coding sequences of human FOXP4 to subclone into the pCAG-mycflag vector, which was modified from pCMV6 Entry (Origene, USA), were also amplified by PCR using primers (Supplementary Table 4). AR_HEC50B cells were seeded in six-well plates at 5 × 10^5^ cells per well. After overnight culture, vectors were employed to transfect cells using FuGene (Promega, USA) according to the manufacturer’s protocol. At 48 h after transfection, 500 μg/mL neomycin (Thermo Fisher Scientific, USA) was added to the cells for 14 days to establish stable cell lines.

### FOXP4 knockdown in cell lines

For knockdown of FOXP4 in human cell lines, we utilized lentivirus particles of MISSION® small hairpin RNA (FOXP4-shRNA2-4: TRCN0000274833, TRCN0000274834, and TRCN0000274832, respectively, nontarget control, NC-shRNA: SHC202V, Sigma, USA). Cells were infected with FOXP4-shRNA (MOI = 20) or NC-shRNA (MOI = 20), and were selected by subsequent incubation for 2 weeks using puromycin.

For the knockdown of FOXP4 in the mouse cell line, we utilized pRNAi-mU6-puro vector (cat no. SORT-A03, Biosettia, USA). The target sequence was designed with BLOCK-iT^TM^ RNAi designer. The vectors for Foxp4-shRNA 1 and 2 and a control vector, which is targeted LacZ, were created with oligo DNA (Supplementary Table 4). Cells were seeded in 6-well plates at 5 × 10^5^ cells per well. After overnight culture, vectors were employed to transfect cells using Lipofectamine 3000 (Invitrogen, USA) according to the manufacturer’s protocol. At 48 h after transfection, 5 μg/mL puromycin (Thermo Fisher, USA) was added to the cells for 7 days to establish stable cell lines.

### Cell proliferation assay

Cells were seeded in 96-well plates at 3 × 10^3^ cells per well. After 24–96 hours of incubation, cell proliferation was determined using Cell Proliferation Reagent WST-1 (Roche, Switzerland) according to the manufacturer’s instructions. When using AR-transfected cells, the medium was replaced with vehicle (0. 1% DMSO) or DHT-mixed phenol red free-medium containing 5% charcoal-stripped FBS, every 24 hours. All experiments were performed in triplicate.

### Wound-healing assay

The treated human endometrial cancer cells were seeded in 24-well plates at 2 × 10^5^ cells per well. After 24 hours incubation, a wound gap was created using SPLscar^TM^ scratcher (SPL-201924, SPL Life Sciences, South Korea). At 0 to 48 hours after scratch, the wound area was calculated using Image J, and then the fold change from the time-point of 0 hours was calculated. When using AR-transfected cells, the medium was replaced with vehicle (0.1% DMSO) or DHT-mixed phenol red-free-medium containing 5% charcoal-stripped FBS every 24 hours. All experiments were performed in triplicate.

### Soft agar colony formation assay

Bacto agar (BD Difco, USA) was suspended in water to a final concentration of 5%, autoclaved, and cooled to 50 °C. This agar solution (300 μL) was added at a 10-fold concentration to DMEM/F12 medium (2.7 mL), to make a final volume of 3 mL/well and an agar concentration of 0.5%. Then, 0.5% agar solution was immediately poured into 6-well plates. Trypsinized human endometrial cells were counted, and 5 × 10^4^ cells were suspended in 100 µL and seeded on the agar. Then, noble agar (BD Difco, USA) was suspended the same as bacto agar to a final concentration of 0.35%, and 2 mL/well of 0.35% agar solution was immediately poured on the bacto agar and cells. When using AR-transfected cells, vehicle or DHT was mixed in each agar solution. After 10 days of incubation, colonies of more than 200 µm in diameter were randomly scored in triplicate.

### Mouse models

The xenograft mouse model was created by subcutaneously injecting human endometrial cancer cells (1 × 10^7^ cells) suspended in 100 µL of serum-free media into female Balb/c nu/nu mice at 8 weeks old. When treating mice with DHT, they were ovariectomized at 7 weeks old and rested for a week. At 8 weeks old, a 1 cm silastic tube (cat no. 100-00 N, Kaneka medical product, Japan) with or without DHT powder (A0462, TCI, Japan) was implanted under the skin. The tumor size was measured every 5 days. The tumor volume was calculated by 0.5 × width (mm) × length^2^ (mm).

*Pten*^ff^/*PR*^cre/+^ and *Pten*^ff^/*LTF*^cre/+^ female mice were utilized as a model of spontaneously developed endometrial carcinoma and a model of spontaneously developed complex atypical hyperplasia, respectively. Six-week-old *Pten*^ff^/*PR*^cre/+^ and 8-week-old *Pten*^ff^/*LTF*^cre/+^ female mice were ovariectomized, and a 1 cm silastic tube (cat no. 100-00 N, Kaneka Medical Product, Japan) with or without DHT powder was implanted under the skin. The uterus was collected 30 days after implanting the silastic tube.

### Chip sequence

To determine AR-DHT-binding regions on genomic DNA, we performed the Chip-assay. Cells at 1.5 × 10^6^ were seeded in a 100-mmφ dish and kept overnight. The CAG-hAR-DYK vector, or an empty vector, which was modified from pcDNA3.1+/c-(K)-DYK (GenScript, USA), was used to transfect cells with GenomeOne GX. The next day, the medium was replaced with vehicle (0. 1% DMSO) or DHT-mixed phenol red free-medium containing 5% charcoal-stripped FBS and kept for 24 hours. After treatment, cells were fixed with 1% formaldehyde for 10 min at room temperature and treated with glycine to quench the formaldehyde. FLAG M2 antibody (Merck, F3165) was used for ChIP analysis. Cells were washed with ice-cold PBS and harvested in PBS. The nuclear fraction was extracted by NP-40 buffer (10 mM Tris-HCl pH 8, 10 mM NaCl, 0.5% NP-40) for 10 min at 4 °C. Cells were pelleted and resuspended in SDS-sharing buffer (5 mM Tris-HCl pH 8, 0.1% SDS, 1 mM EDTA) and sonicated with a Covaris socinicator. The lysate was centrifuged for 20 min at 14,000 rpm to remove debris. The lysate was then diluted in ChIP dilution buffer (50 mM Tris-HCl pH 8, 167 mM NaCl, 1.1% Triton X-100, 0.11% sodium deoxycholate). The sample was incubated with 20 uL of Dynabeads of anti-mouse IgG conjugated with anti-FLAG M2 antibody overnight at 4 °C. The beads were then washed in low-salt RIPA buffer (20 mM Tris-HCl pH 8, 150 mM NaCl, 1 mM EDTA, 0.1% sodium deoxycholate and 1% SDS) for 5 min at 4 °C, high-salt RIPA buffer (50 mM Tris-HCl pH 8, 500 mM NaCl, 1 mM EDTA, 0.1% sodium deoxycholate and 1% SDS) for 5 min at 4 °C, LiCl wash buffer (10 mM Tris-HCl pH 8, 250 mM LiCl, 1 mM EDTA, 0.5% sodium deoxycholate and 0.5% NP-40) for 5 min at 4 °C, and TE for 5 min at 4 °C. DNA was de-crosslinked and eluted in elution buffer (10 mM Tris-HCl pH 8, 300 mM NaCl, 5 mM EDTA, and 0.5% SDS) overnight at 65 °C. RNA and protein were digested with 0.2 mg/mL RNase A for 30 min at 37 °C and then incubated with 0.2 mg/mL Proteinase K for 2 h at 55 °C. DNA was purified using QIAquick PCR Purification Kit. Sequencing libraries were prepared from the ChIP and Input DNAs using SMARTer ThruPLEX Tag-seq Kit (Clontech) according to the manufacturer’s protocol. Single-read sequencing (75 bp) was performed on the Illumina NextSeq 500 sequencer. The trimming adaptor sequence and 7–17 bp of the 5’ end of reads after removing duplicate reads were assessed using Seqkit (ver.0.10.2). Trimmed reads were mapped to the mm10 reference sequence using Burrows–Wheeler Aligner (BWA-MEM: ver. 0.7.17-r1188), and duplicate reads were removed with Picard (ver. 2.18.16). Peak calling was performed using PePr (ver. 1.1.24), MACS2 (ver. 2.1.2), and epic2 (ver. 0.0.40) with default parameters, respectively. Peak annotations were conducted by HOMER (ver. 4.9.1) with default settings. Known motifs and de novo consensus motifs in the central 200 bp of obtained peaks were searched by HOMER with default settings.

### CRISPR/Cas9

To delete the AR-DHT binding site in the h*FOXP4* coding region, pX330 vector with L-gRNA or R-gRNA (Supplementary Table 4) was created. Then, 1.5 × 10^6^ AR_HEC50B and CAG_HEC50B cells were seeded in a 100-mmφ dish and kept overnight. pX330-L-gRNA and -R-gRNA were used to transfect cells with GenomeOne GX. From the day after transfection, the medium was replaced every 24 hours with vehicle (0. 1% DMSO) or DHT-mixed phenol red free-medium containing 5% charcoal-stripped FBS and kept for 48 hours. RNA and genomic DNA were extracted with Trizol (Invitrogen). The deletion of genomic DNA was determined by PCR with primers (Supplementary Table 4).

### Statistics and reproducibility

Statistical analyses were carried out using SPSS Statistics version 25.0 (IBM, USA). We utilized the *χ*^2^ test, Kaplan–Meier method, log-rank test, Kruskal–Wallis test, Mann–Whitney *U* test, and correlation analysis; ANOVA was followed by the Bonferroni test or Dunnett test depending on the experiment. *P* values < 0.05 were considered significant in this study. The sample size followed standards in the field. All attempts for data replication were successful. The details were explained in the Methods section and figure legend.

### Reporting summary

Further information on research design is available in the Nature Portfolio Reporting Summary linked to this article.

### Supplementary information

Supplementary Information

Description of Additional Supplementary Files

Supplementary data 1

Reporting Summary

## Data Availability

Nucleotide sequence data reported are available in the DDBJ Sequenced Read Archive under the accession number DRA016966. Processed data are available in DDBJ Genomic Expression Archive under the accession number E-GEAD-637. Full western blot and DNA gel electrophoresis images are shown in Supplementary Fig. 7. The source data behind the graphs in the paper can be found in Supplementary Data 1.

## Code availability

Burrows-Wheeler Aligner (BWA-MEM: ver. 0.7.17-r1188) and Picard (ver. 2.18.16) are used for data collection. PePr (ver. 1.1.24), MACS2 (ver. 2.1.2), epic2 (ver. 0.0.40), HOMER (ver. 4.9.1), HOMER, and SPSS Statistics version 25.0 are used for data analysis. The detailed information is written in the Methods section of the paper.
