# Supplementary material for: Androgen-responsive FOXP4 is a target for endometrial carcinoma
Source: Commun Biol. 2024 Jun 18;7:740. doi: 10.1038/s42003-024-06433-w (PMC11189448; doi:10.1038/s42003-024-06433-w)

Supplementary Table 1. Clinicopathological characteristics of endometrial carcinoma

	AR		
Variable	Low	High	Pvalue
Age, mean ± SD (years)	59.9 ± 12.0	62.3 ± 10.5	0.144
BMI, mean ± SD	23.9 ± 4.8	24.4 ± 5.0	0.464
Parity (no.)			0.288
0	40	16	
1 o more	187	51	
CA125, mean ± SD (U/mL)	43.0 ± 84.5	40.8 ± 83.3	0.852
Stage (no.)			0.171
Stage Ⅰ/Ⅱ	189	61	
Stage Ⅲ/Ⅳ	38	6	
Grade (no.)			0.0161*
1	124	48	
2・3	103	19	
Myometrial invasion (no.)			0.189
>1/2	81	18	
≤1/2	146	49	
Lymph node metastasis (no.)			0.0899
Positive	32	4	
Negative	195	63	
Distant site metastasis (no.)			1
Positive	5	1	
Negative	222	66	
FOXP4 expression (no.)			0.0298*
High	87	16	
Low	140	51	

Statistical analysis was performed with $\chi 2$ test. The asterisk showed P value was significantly different.

Supplementary Table 2. Univariate and multivariate analysis of predictive factors for survival in endometrial carcinoma

		Disease free survival		Overall survival	
Factors	n	Univariate P value	Multivariate P value	Univariate P value	Multivariate P value
Age (years)		$1.20e^{-4}$*		.0350*	
≤60	157				
>60	137				
BMI		0.949		0.785	
≤23	154				
>23	240				
Parity		0.131		0.0646	
0	56				
1 or more	238				
CA125		0.0159*		.00405*	
≤35	215				
>35	68				
NA	11				
Stage		$2.91e^{-10}$*	.0244*	$3.47e^{-4}$*	0.208
Stage Ⅰ / Ⅱ	250				
Stage Ⅲ/Ⅳ	44				
Grade		0.12	0.511	0.319	0.593
1	172				
2・3	122				
Myometrial invasion (no.)		$3.12e^{-7}$*	.00499*	.00263*	.0161*
>1/2	98				
≤1/2	196				
Lymph node metastasis (no.)		$2.49e^{-11}$*		$3.62e^{-5}$*	
Positive	36				
Negative	258				
Distant site metastasis (no.)		$8.40e^{-5}$*	0.426	7.82e-9*	.0332*
Positive	5				
Negative	289				
FOXP4 expression (no.)		$2.06e^{-7}$*	$5.10e^{-5}$*	.0296*	0.181
High	77				
Low	217				
AR expression(no.)		.0192*	0.0915	0.345	0.771
High	67				
Low	227				

Statistical analysis was performed with Cox Regression Analysis. The asterisk showed P value was significantly different.

Supplementary Table 3. AR and FOXP4 gene mutation statuses in human ECCs

Cell line	Gene mutation	
	AR	FOXP4
HEC265	No alteration	No alteration
HEC59	Q78*	No alteration
HEC50B	V714A	No alteration
HEC108	Q78*, M247V, D691del	No alteration

Supplementary Table 4. Oligo DNA sequences

Purpose	Oligo DNA
h*FOXP4* primers for qPCR	Forward: CCAGGATGTTCGCCTATTTC
	Reverse: TTGTGCAGGCTGAGGTTGT
h*GAPDH* primers for qPCR	Forward: GCACCGTCAAGGCTGAGAAC
	Reverse: TGGTGAAGACGCCAGTGGA
h*FOXP4* primers for amplifying cDNA	Forward: AAACTCGAGTAGAGCGACATGATGGTGGA
	Reverse: TTTCTCGAGCCTTAGGACAGTTCTTCTCCCG
m*FoxpP4* primers for amplifying cDNA	Forward: ATGATGGTGGAGTCTGCATC
	Reverse: TTAGGACATGTCCTCTCCTC
hFOXP4 primer for subcloning to pCAG-mycflag	Forward: GATCGCCGGCGCGCCAGATCTATGATGGTGGAATCTGCCTC
	Reverse: TTTCTGCTCGAGCGGCCGCGTGGACAGTTCTTCTCCCGGCA
m*Foxp4* -shRNA 1	AAAAGCAGGAGAAGTAATGACAAATTTGGATCCAA
	ATTTGTCATTACTTCTCCTGC
m*Foxp4* -shRNA 2	AAAAGCTGACGCTAAATGAGATTTATTGGATCCAAT
	AAATCTCATTTAGCGTCAGC
lacZ-shRNA	AAAAGCAGTTATCTGGAAGATCAGGTTGGATCCAA
	CCTGATCTTCCAGATAACTGC
h*FOXP4* intron 1 targeting L-gRNA	CCCTGGTTTCATTTCACACG
h*FOXP4* intron 1 targeting R-gRNA	GGTTAGGTCTTTGTACCATA
h*FOXP4* genetic DNA detecting primers	Forward: ACATGCAACTTGGAAAATCAGAAAT
	Reverse: AAGAAAGAGAACCAAAGATCCCACT

Supplementary Figure 1

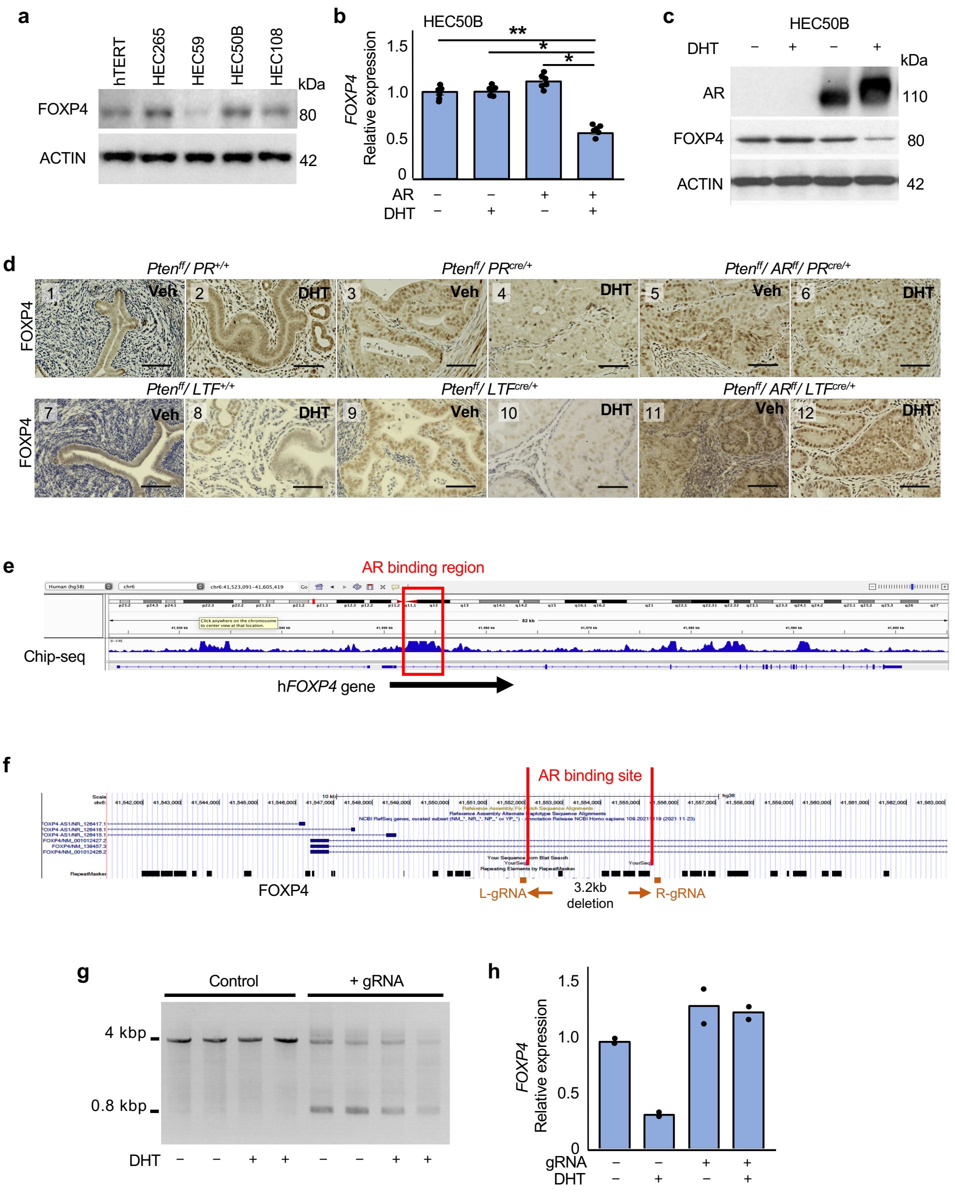

Supplementary Figure 1. The downstream target molecule of androgen/AR pathway

(a) FOXP4 expression in an immortalized endometrial epithelial cell line (hTERT) and endometrial cancer cell lines (HEC265, HEC59, HEC50B, HEC108). ACTIN was used as an internal control. **(b & c)** The expression of FOXP4 mRNA (B) and protein (C) in AR_HEC50B cells was suppressed with DHT (10 nM) treatment (72 hours) but not with vehicle treatment (0.1 % DMSO). **(d)** The FOXP4 expression in *Pten* deleted mice was reduced with DHT treatment (n=3). Bars show 100 µm. **(e)** Androgen/AR binding region on human FOXP4 coding region. **(f)** The deleted region on hFOXP4 coding region by CRISPR/Cas9. **(g)** The deletion was detected with genomic DNA by PCR. **(h)** The FOXP4 expression was detected by qPCR (n = 2). DHT (-); 0.1 % DMSO, DHT (+); 10 nM. Statistical analysis was performed by ANOVA followed by the Bonferroni test. Error bars represent standard error. *$p<0.05$, **$p<0.01$.

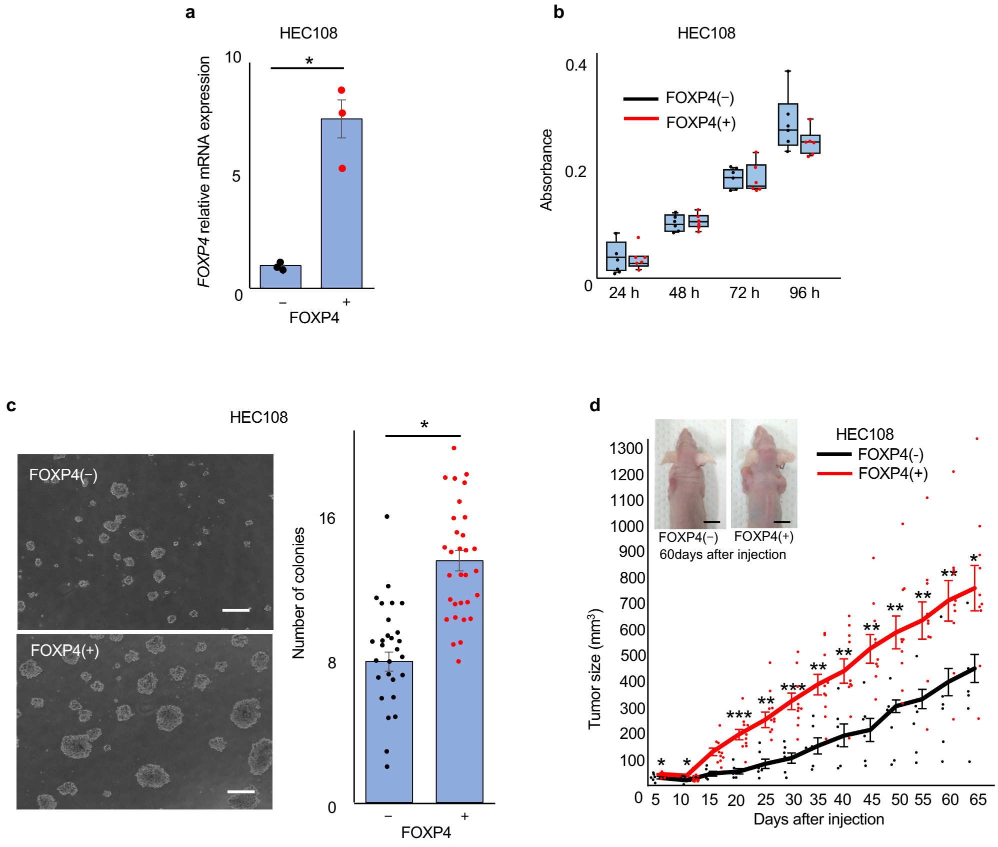

Supplementary Figure 2. The effect of FOXP4 overexpression on HEC108 cells
(a) HEC108 cells were transfected with an empty vector (FOXP4(-)) or a FOXP4 overexpression vector. (FOXP4(+)). **(b)** Cell proliferation was not increased by FOXP4 overexpression (n=6). **(c)** Colony-forming ability was increased by FOXP4 overexpression (n=30). Bars show 200 µm. **(d)** Tumor growth of FOXP4(+)HEC108 cells was increased compared to FOXP4(-)HEC108 cells (n=6). Bars show 1 cm. Statistical analysis was performed by the Mann-Whitney U test. Error bars represent standard error. *p<0.05, **p<0.01, ***p<0.001.

Supplementary Figure 3

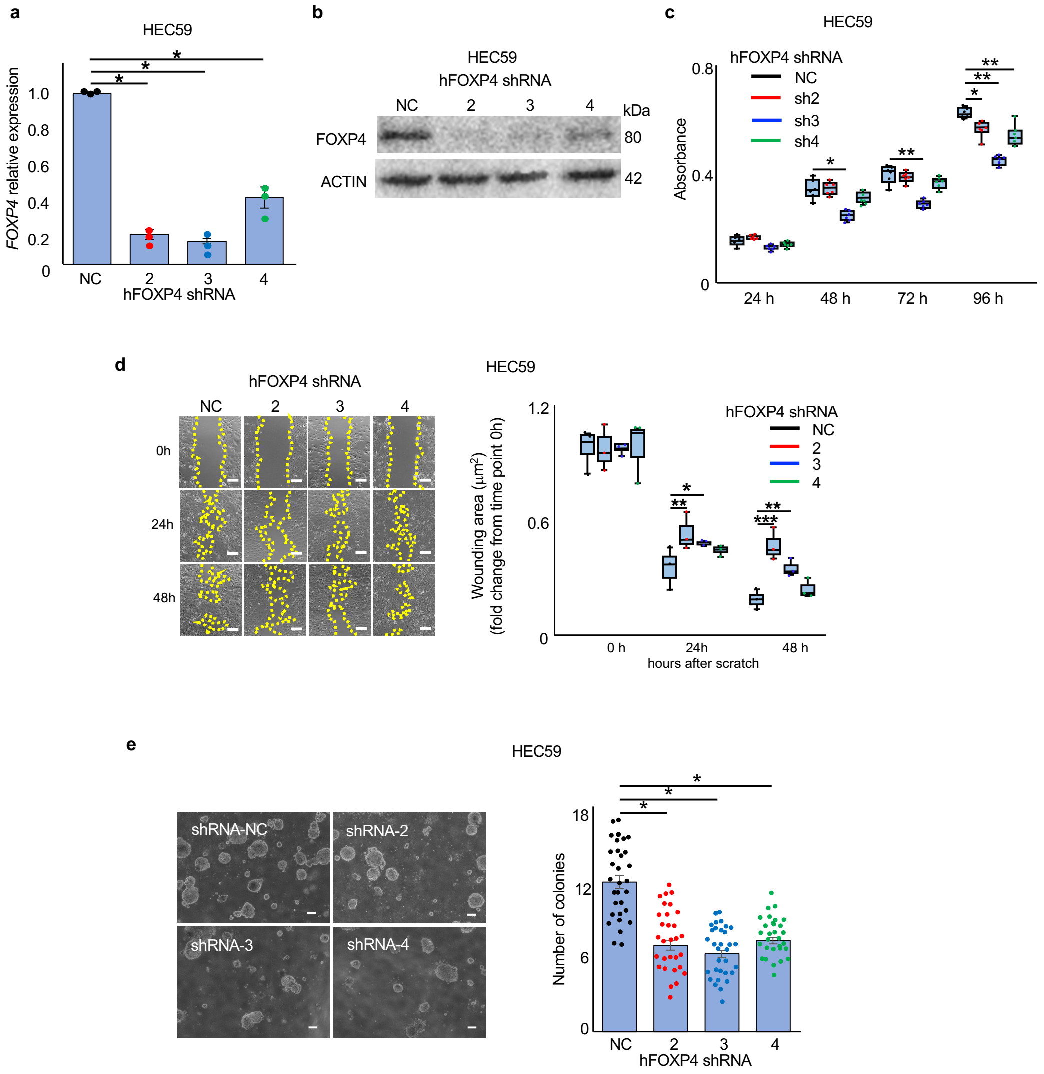

Supplementary Figure 3. The effect of FOXP4 knockdown on HEC59 cells
(a & b) The expression level of FOXP4 mRNA **(a)** and protein **(b)**. HEC59 cells were infected with lentivirus carrying shRNA of FOXP4 or non-target control. ACTIN served as an internal control. **(c)** Cell proliferation showed a significant difference between HEC59 cells with FOXP4 shRNA and non-target control shRNA (n=6). **(d)** Wound healing ability was decreased by FOXP4 knockdown (n = 3). **(e)** Colony-forming ability was decreased by FOXP4 knockdown (n = 30). Bars show 200μm **(d and e)**. Statistical analysis was performed by ANOVA followed by the Dunnett test. Error bars represent standard error. *p<0.05, **p<0.01, ***p<0.001.

Supplementary Figure 4

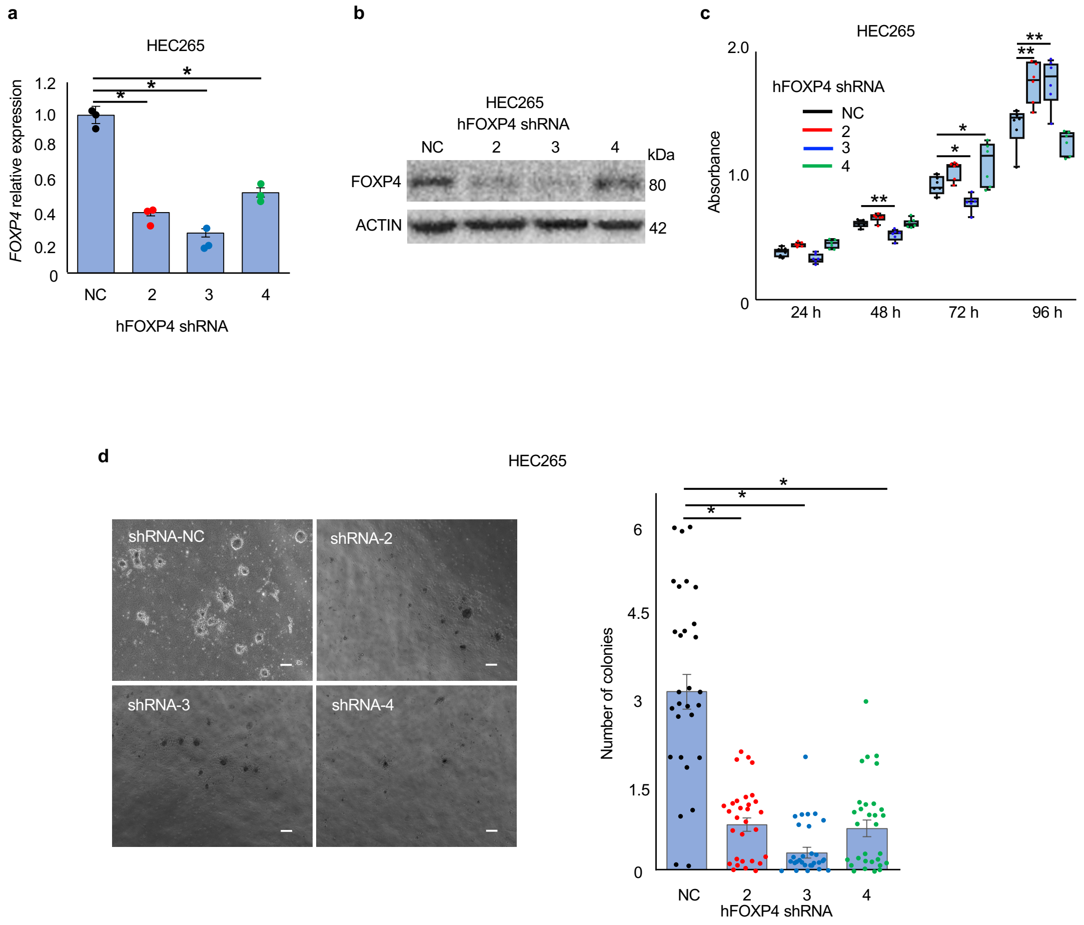

Supplementary Figure 4. The effect of FOXP4 knockdown on HEC265 cells
(a & b) The expression level of FOXP4 mRNA **(a)** and protein **(b)**. HEC265 cells were infected with lentivirus carrying shRNA of FOXP4 or non-target control. ACTIN served as an internal control. **(c)** Cell proliferation showed a significant difference between HEC265 cells with FOXP4 shRNA and non-target control shRNA (n=6). **(d)** Colony-forming ability was decreased by FOXP4 knockdown (n=30). Bars show 200 µm. Statistical analysis was performed by ANOVA followed by the Dunnett test. Error bars represent standard error. *p<0.05, **p<0.01.

Supplementary Figure 5

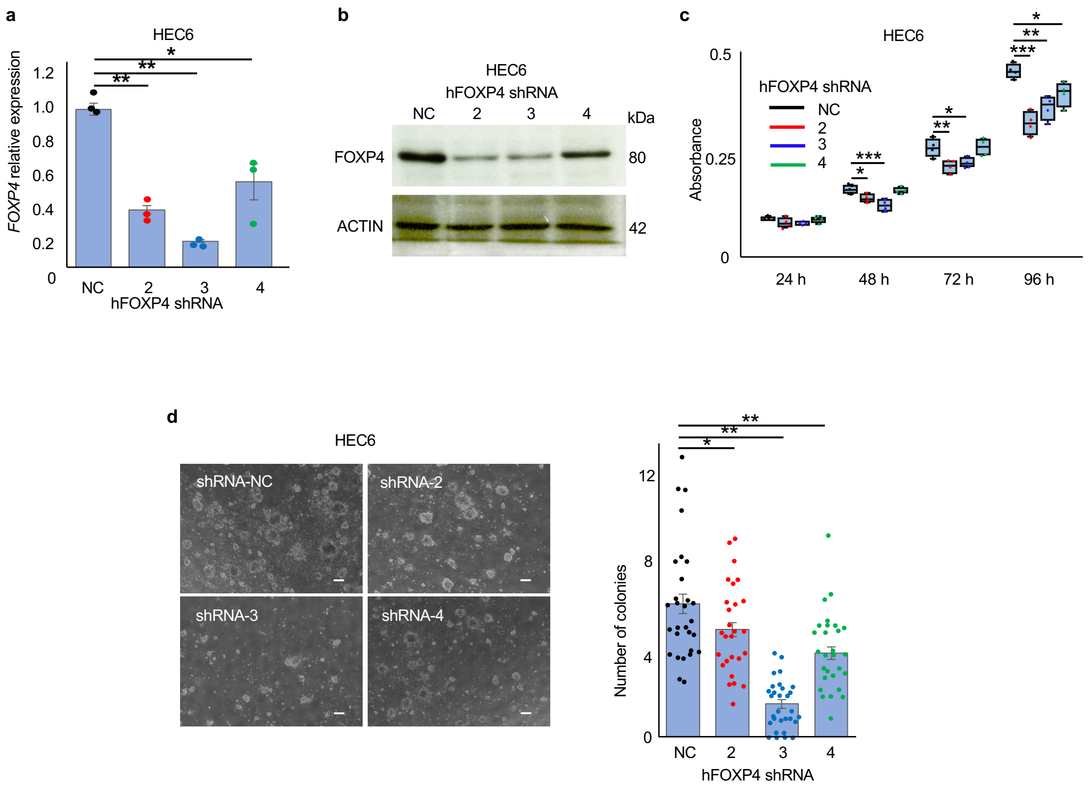

Supplementary Figure 5. The effect of FOXP4 knockdown on HEC6 cells
(a & b) The expression level of FOXP4 mRNA **(a)** and protein **(b)**. HEC6 cells were infected with lentivirus carrying shRNA of FOXP4 or non-target control. ACTIN served as an internal control. **(c)** Cell proliferation showed a significant difference between HEC6 cells with FOXP4 shRNA and non-target control shRNA (n=4). **(d)** Colony-forming ability was decreased by FOXP4 knockdown (n = 30). Bars show 200 µm. Statistical analysis was performed by ANOVA followed by the Dunnett test. Error bars represent standard error. *$p<0.05$, **$p<0.01$, ***$p<0.001$.

Supplementary Figure 6

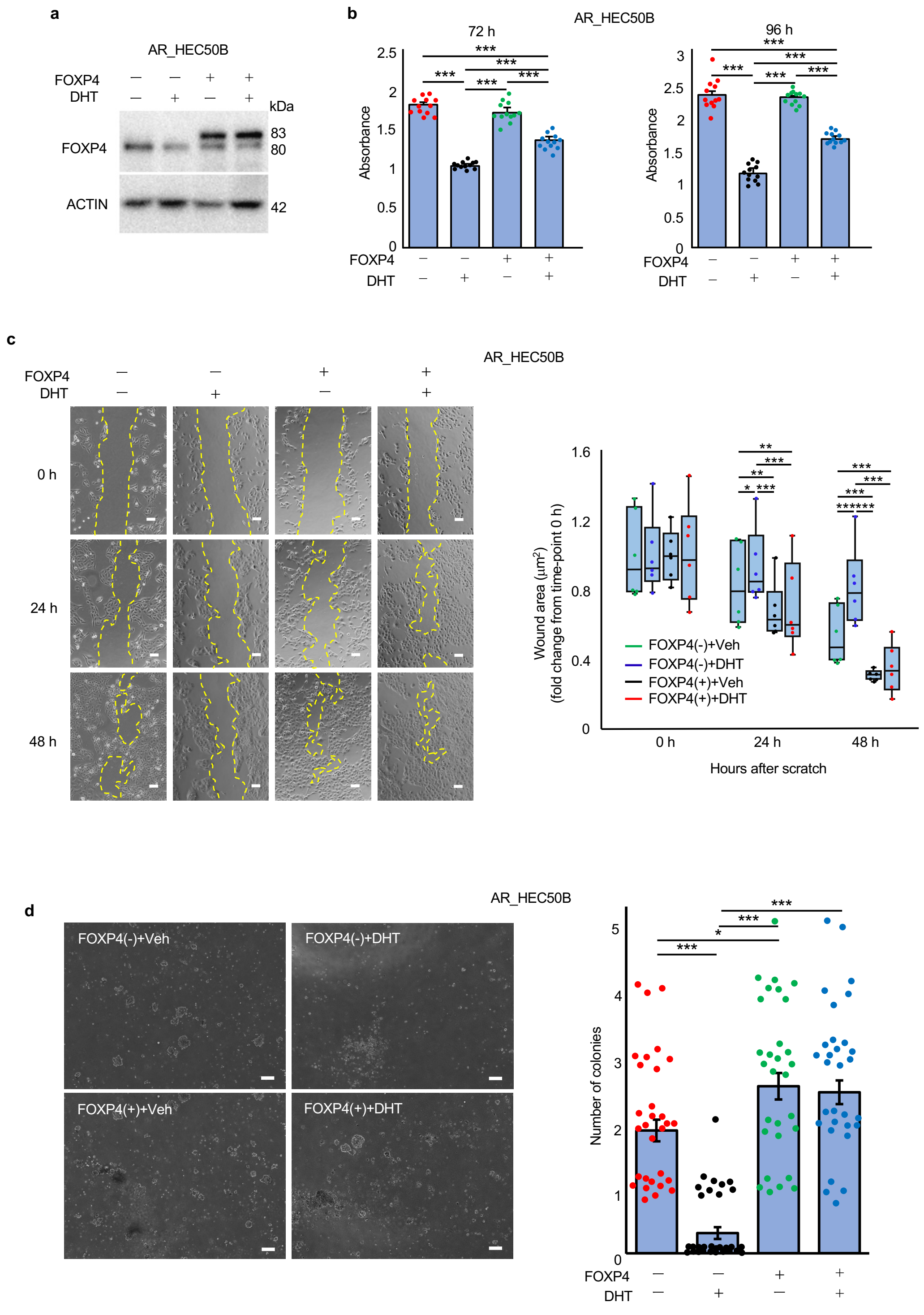

Supplementary Figure 6. The effect of FOXP4 overexpression on AR_HEC50B cells
(a) AR_HEC50B was transfected with FOXP4-mycflag overexpression or empty vector. **(b)** Endogenous FOXP4 partially restores the effect of 10 nM DHT treatment on cell viability. **(c & d)** Endogenous FOXP4 completely restores the effects of 10 nM DHT treatment on wound healing (n=3) **(c)** and colony-forming ability **(d)** in AR_HEC50B cells. Bars show 200 μm. Statistical analysis was performed by ANOVA followed by the Bonferroni test. Error bars represent standard error. *p<0.05, **p<0.01, ***p<0.001.

Supplementary Figure 7

Figure 2a

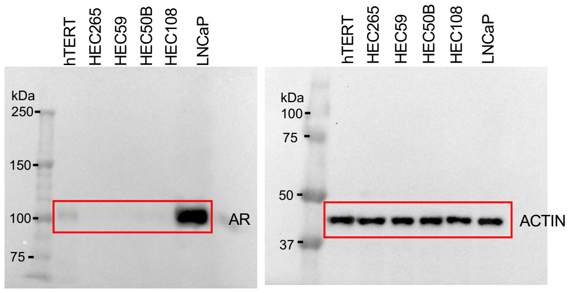

Figure 2b

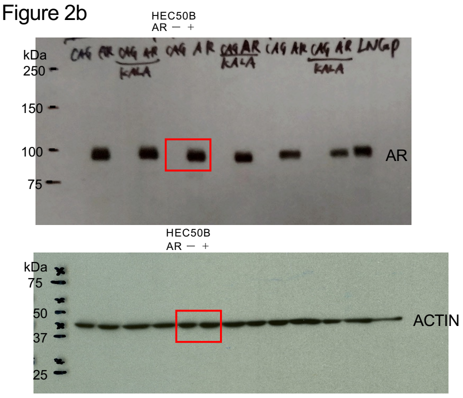

Figure 3b

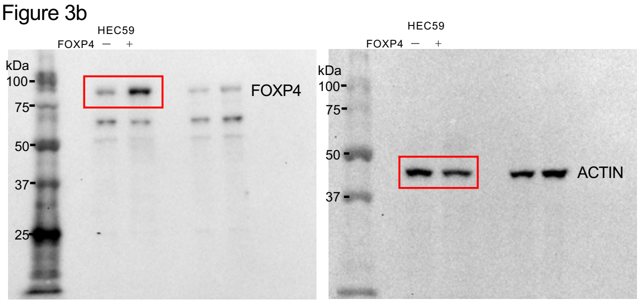

Figure 3f

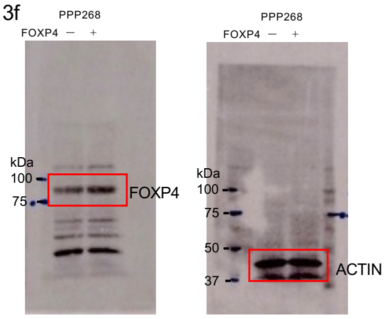

Figure 4b

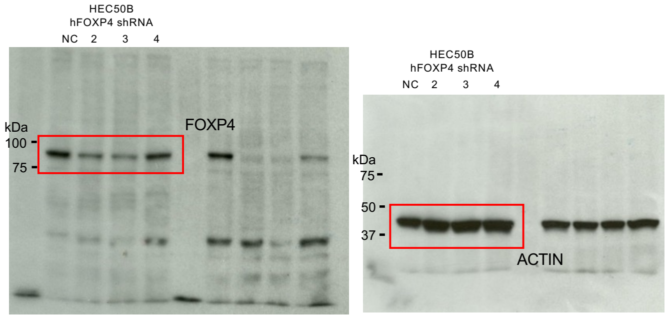

Figure 4g

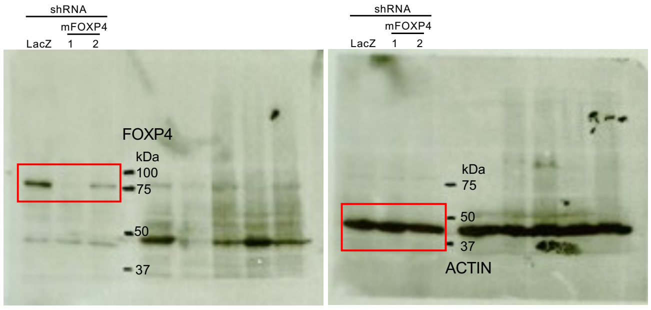

Supplementary Figure 1a

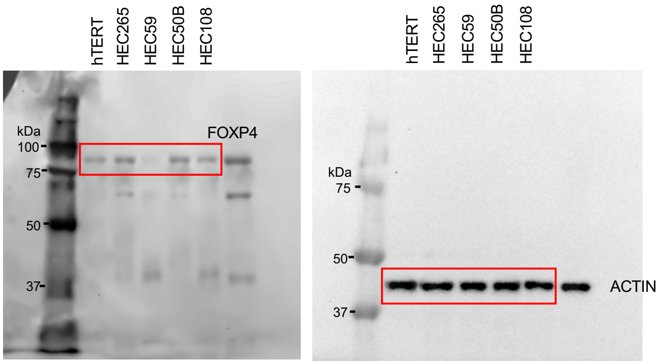

Supplementary Figure1c

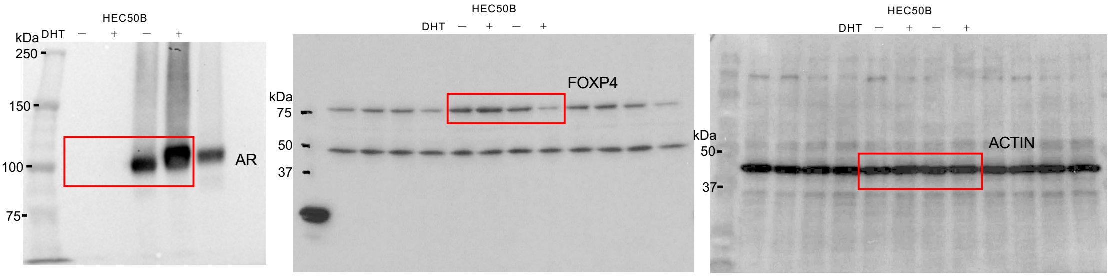

Supplementary Figure 7 (continued)

Supplementary Figure1g

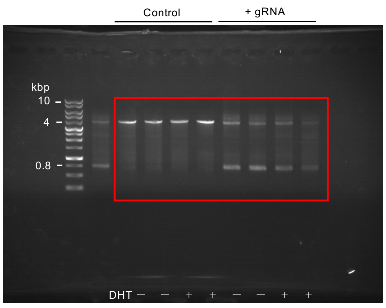

Supplementary Figure 3b

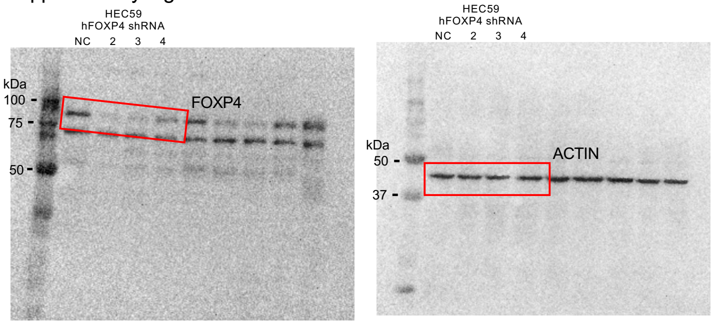

Supplementary Figure 4b

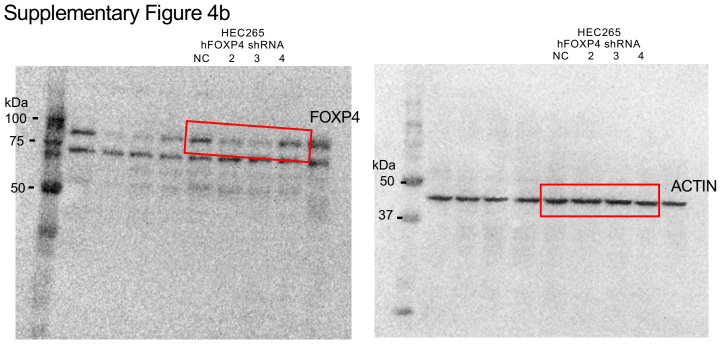

Supplementary Figure 5b

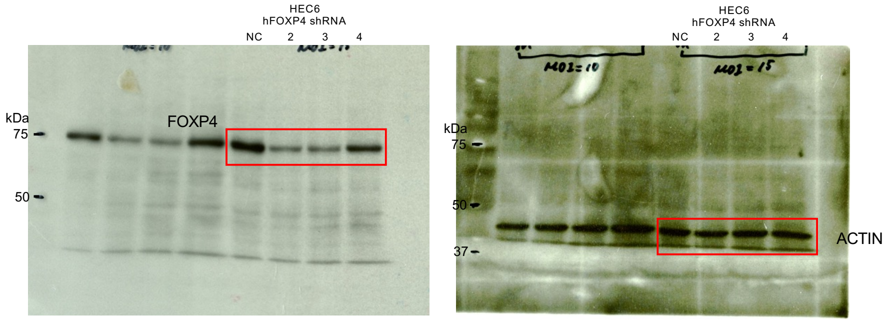

Supplementary Figure 6a

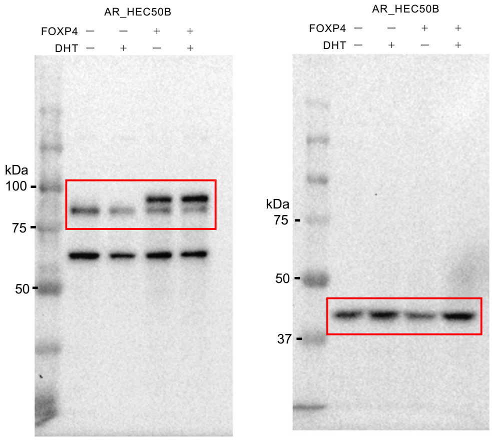

Supplement: Supplementary file 1 — Supplementary Information [file 42003_2024_6433_MOESM1_ESM.pdf]
