# Supplementary material for: Androgen-responsive FOXP4 is a target for endometrial carcinoma
Source: Commun Biol. 2024 Jun 18;7:740. doi: 10.1038/s42003-024-06433-w (PMC11189448; doi:10.1038/s42003-024-06433-w)
Supplement: Supplementary file 4 — Reporting Summary [file 42003_2024_6433_MOESM4_ESM.pdf]

nature portfolio

Corresponding author(s): Takiko Daikoku

Last updated by author(s): 5/8/2024

Reporting Summary

Nature Portfolio wishes to improve the reproducibility of the work that we publish. This form provides structure for consistency and transparency in reporting. For further information on Nature Portfolio policies, see our Editorial Policies and the Editorial Policy Checklist.

Statistics

For all statistical analyses, confirm that the following items are present in the figure legend, table legend, main text, or Methods section.

n/a	Confirmed	
☐	☒	The exact sample size (n) for each experimental group/condition, given as a discrete number and unit of measurement
☐	☒	A statement on whether measurements were taken from distinct samples or whether the same sample was measured repeatedly
☐	☒	The statistical test(s) used AND whether they are one- or two-sided<br>*Only common tests should be described solely by name; describe more complex techniques in the Methods section.*
☐	☒	A description of all covariates tested
☐	☒	A description of any assumptions or corrections, such as tests of normality and adjustment for multiple comparisons
☐	☒	A full description of the statistical parameters including central tendency (e.g. means) or other basic estimates (e.g. regression coefficient) AND variation (e.g. standard deviation) or associated estimates of uncertainty (e.g. confidence intervals)
☐	☒	For null hypothesis testing, the test statistic (e.g. F, t, r) with confidence intervals, effect sizes, degrees of freedom and P value noted<br>*Give P values as exact values whenever suitable.*
☒	☐	For Bayesian analysis, information on the choice of priors and Markov chain Monte Carlo settings
☐	☒	For hierarchical and complex designs, identification of the appropriate level for tests and full reporting of outcomes
☒	☐	Estimates of effect sizes (e.g. Cohen's d, Pearson's r), indicating how they were calculated

Our web collection on statistics for biologists contains articles on many of the points above.

Software and code

Policy information about availability of computer code

Data collection	Burrows-Wheeler Aligner (BWA-MEM: ver. 0.7.17-r1188), Picard (ver. 2.18.16)
Data analysis	PePr (ver. 1.1.24), MACS2 (ver. 2.1.2), epic2 (ver. 0.0.40), HOMER (ver. 4.9.1), HOMER, SPSS Statistics version 25.0

For manuscripts utilizing custom algorithms or software that are central to the research but not yet described in published literature, software must be made available to editors and reviewers. We strongly encourage code deposition in a community repository (e.g. GitHub). See the Nature Portfolio guidelines for submitting code & software for further information.

Data

Policy information about availability of data

All manuscripts must include a data availability statement. This statement should provide the following information, where applicable:

- Accession codes, unique identifiers, or web links for publicly available datasets
- A description of any restrictions on data availability
- For clinical datasets or third party data, please ensure that the statement adheres to our policy

Accession number for Chip-seq data is PRJDB16447 in DDBJ.

Research involving human participants, their data, or biological material

Policy information about studies with human participants or human data. See also policy information about sex, gender (identity/presentation), and sexual orientation and race, ethnicity and racism.

Reporting on sex and gender	female
Reporting on race, ethnicity, or other socially relevant groupings	Japanese
Population characteristics	endometrial cancer patients
Recruitment	We obtained the cooperation of patients who consented among the patients who nderwent surgery.
Ethics oversight	Kanazawa University

Note that full information on the approval of the study protocol must also be provided in the manuscript.

Field-specific reporting

Please select the one below that is the best fit for your research. If you are not sure, read the appropriate sections before making your selection.

☒ Life sciences ☐ Behavioural & social sciences ☐ Ecological, evolutionary & environmental sciences

For a reference copy of the document with all sections, see nature.com/documents/nr-reporting-summary-flat.pdf

Life sciences study design

All studies must disclose on these points even when the disclosure is negative.

Sample size	We followed standards in the field.
Data exclusions	The outliers were excluded by the Smirnov-Grubbs test after serum concentrations of DHT were measured by ELISA.
Replication	All attempts for data replication were successful.
Randomization	Randomization was applied when possible, such as tumor growth assay with nude mice.
Blinding	Blinding was applied when possible, such as immunohistochemistry of human tissue.

Reporting for specific materials, systems and methods

We require information from authors about some types of materials, experimental systems and methods used in many studies. Here, indicate whether each material, system or method listed is relevant to your study. If you are not sure if a list item applies to your research, read the appropriate section before selecting a response.

Materials & experimental systems

n/a	Involved in the study	
☐	☒	Antibodies
☐	☒	Eukaryotic cell lines
☒	☐	Palaeontology and archaeology
☐	☒	Animals and other organisms
☒	☐	Clinical data
☒	☐	Dual use research of concern
☒	☐	Plants

Methods

n/a	Involved in the study	
☐	☒	ChIP-seq
☒	☐	Flow cytometry
☒	☐	MRI-based neuroimaging

Antibodies

Antibodies used	For IHC<br>anti-AR rabbit monoclonal antibody (clone SP107, RRID: AB_2537931, ab105225, Abcam, USA), anti-FOXP4 rabbit polyclonal antibody (HPA007176, Sigma, USA), and anti-Ki67 rabbit monoclonal antibody (clone sp6, RM-9106, Thermo Fisher, USA)<br>For WB

	antibody against AR (clone SP107, RRID: AB_2537931, ab105225, Abcam, USA), or rabbit polyclonal antibody which was kindly gifted from Dr. Mizokami), FOXP4 (RRID: AB_2262825, 16772-1-AP, Proteintech Group Inc., USA), and β-Actin (RRID: AB_630835, C-11, Santa Cruz Biotechnology, USA)
Validation	https://www.abcam.co.jp/products/primary-antibodies/androgen-receptor-antibody-sp107-n-terminal-ab105225.html<br>https://www.sigmaaldrich.com/JP/ja/product/sigma/hpa007176<br>https://www.thermofisher.com/antibody/product/Ki-67-Antibody-clone-SP6-Recombinant-Monoclonal/MA5-14520<br>https://www.ptgcn.com/Products/FOXP4-Antibody-16772-1-AP.htm<br>https://www.scbt.com/ja/p/actin-antibody-c-11<br>We published data with FOXP4 and AR antibodies in Cancer Med. 2023 May;12(9):10816-10828. doi: 10.1002/cam4.5824. Epub 2023 Mar 23.

Eukaryotic cell lines

Policy information about cell lines and Sex and Gender in Research

Cell line source(s)	The human endometrial carcinoma cell lines, HEC265, HEC59, HEC50B, and HEC108, generated by Dr. Hiroyuki Kuramoto were kindly provided by Dr. Katsutoshi Oda (Graduate School of Medicine, Tokyo University). The human AR-positive prostate cancer cell line, LNCaP, was gifted by Dr. Atsushi Mizokami (Department of Urology, Graduate School of Medical Sciences, Kanazawa University). An immortalized human endometrial epithelial cell line, EM-E6/E7/hTERT cells, was previously established in OB/GYN in Kanazawa University. The mouse endometrial cancer cell line, PPP268, was established by us.
Authentication	All human cell lines were authenticated by STR profiling.
Mycoplasma contamination	Mycoplasma infections were regularly monitored and all experiments were performed under mycoplasma-free conditions.
Commonly misidentified lines<br>(See ICLAC register)	The mouse endometrial cancer cell line, PPP268, was established by us.

Animals and other research organisms

Policy information about studies involving animals; ARRIVE guidelines recommended for reporting animal research, and Sex and Gender in Research

Laboratory animals	Mice, C57BL/6, Pten ff/PRcre, Pten ff/Ltfcre, Balb/c nu/nu, 6 to 12 weeks old females
Wild animals	The study does not involve wild animals.
Reporting on sex	Female
Field-collected samples	The study does not involve samples collected from field.
Ethics oversight	Kanazawa University

Note that full information on the approval of the study protocol must also be provided in the manuscript.

Plants

Seed stocks	n/a
Novel plant genotypes	n/a
Authentication	n/a

ChIP-seq

Data deposition

☒ Confirm that both raw and final processed data have been deposited in a public database such as GEO.

☒ Confirm that you have deposited or provided access to graph files (e.g. BED files) for the called peaks.

Data access links<br>*May remain private before publication.*	https://ddbj.nig.ac.jp/public/ddbj_database/gea/experiment/E-GEAD-000/E-GEAD-637
Files in database submission	AR.10.10per.input_AR.10.10per.input_R1.fastq<br>AR.10.10per.input_AR.10.10per.input_R2.fastq<br>AR.10_AR.10_R1.fastq<br>AR.10_AR.10_R2.fastq

GFP.10.10per.input_GFP.10.10per.input_R1.fastq
GFP.10.10per.input_GFP.10.10per.input_R2.fastq
GFP.10_GFP.10_R1.fastq
GFP.10_GFP.10_R2.fastq
AR.10.10per.input-AR.10_PeakCall.bed
GFP.10.10per.input-GFP.10_PeakCall.bed
AR.10.10per.input-AR.10_PeakCall_annotated.txt
GFP.10.10per.input-GFP.10_PeakCall_annotated.txt
AR.10_AR.10.bw
GFP.10_GFP.10.bw

Genome browser session (e.g. UCSC): UCSC

Methodology

Replicates: n=1

Sequencing depth: 80 million

Antibodies: mouse anti-FLAG M2

Peak calling parameters: Default

Data quality: QC passed

Software: Python
